# Identification of influential weather parameters and seasonal drought prediction in Bangladesh using machine learning algorithm

**DOI:** 10.1038/s41598-023-51111-2

**Published:** 2024-01-04

**Authors:** Md. Abdullah Al Mamun, Mou Rani Sarker, Md Abdur Rouf Sarkar, Sujit Kumar Roy, Sheikh Arafat Islam Nihad, Andrew M. McKenzie, Md. Ismail Hossain, Md. Shahjahan Kabir

**Affiliations:** 1 Agricultural Statistics Division, Bangladesh Rice Research Institute, Gazipur, 1701 Bangladesh; 2 Sustainable Impact Platform, International Rice Research Institute, Dhaka, 1213 Bangladesh; 3 School of Economics, Zhongnan University of Economics and Law, Wuhan, 430073 China; 4 Agricultural Economics Division, Bangladesh Rice Research Institute, Gazipur, 1701 Bangladesh; 5 Institute of Water and Flood Management, Bangladesh University of Engineering and Technology, Dhaka, 1000 Bangladesh; 6 Plant Pathology Division, Bangladesh Rice Research Institute, Gazipur, 1701 Bangladesh; 7 Department of Agricultural Economics and Agribusiness, The University of Arkansas, Fayetteville, AR 72701 USA; 8 Bangladesh Rice Research Institute, Gazipur, 1701 Bangladesh

**Keywords:** Climate sciences, Environmental sciences, Hydrology, Natural hazards

## Abstract

Droughts pose a severe environmental risk in countries that rely heavily on agriculture, resulting in heightened levels of concern regarding food security and livelihood enhancement. Bangladesh is highly susceptible to environmental hazards, with droughts further exacerbating the precarious situation for its 170 million inhabitants. Therefore, we are endeavouring to highlight the identification of the relative importance of climatic attributes and the estimation of the seasonal intensity and frequency of droughts in Bangladesh. With a period of forty years (1981–2020) of weather data, sophisticated machine learning (ML) methods were employed to classify 35 agroclimatic regions into dry or wet conditions using nine weather parameters, as determined by the Standardized Precipitation Evapotranspiration Index (SPEI). Out of 24 ML algorithms, the four best ML methods, ranger, bagEarth, support vector machine, and random forest (RF) have been identified for the prediction of multi-scale drought indices. The RF classifier and the Boruta algorithms shows that water balance, precipitation, maximum and minimum temperature have a higher influence on drought intensity and occurrence across Bangladesh. The trend of spatio-temporal analysis indicates, drought intensity has decreased over time, but return time has increased. There was significant variation in changing the spatial nature of drought intensity. Spatially, the drought intensity shifted from the northern to central and southern zones of Bangladesh, which had an adverse impact on crop production and the livelihood of rural and urban households. So, this precise study has important implications for the understanding of drought prediction and how to best mitigate its impacts. Additionally, the study emphasizes the need for better collaboration between relevant stakeholders, such as policymakers, researchers, communities, and local actors, to develop effective adaptation strategies and increase monitoring of weather conditions for the meticulous management of droughts in Bangladesh.

## Introduction

Climate change has had and continues to have catastrophic effects on humanity. Severe weather occurrences, particularly heat waves, droughts, cyclones, and heavy rain, are becoming more frequent and intense, leading to displacement, famine, and poverty^1^. Drought, the most frequent climate occurrence worldwide, is characterized by a shortage of precipitation which causes long-term water scarcities^2–5^. Droughts are one of the most expensive calamities, affecting millions of people annually and costing an estimated $6 to $8 billion annually^6^. However, the slow-onset nature of drought makes it challenging to analyze and model its spatio-temporal consequences.

Bangladesh is one of the utmost prone to natural catastrophes nations in the biosphere because of its geographical location^7^. Drought is a frequent natural disaster in the country. Bangladesh experienced extreme droughts in 1973, 1978, 1979, 1981, 1982, 1992, 1994, 1995, 2000, 2006, and 2009^8^. Scholars recognized that drought poses a significant risk to food security^6,9,10^. Climate change and scarcity of groundwater, combined with unpredictable rainfall and high temperatures, negatively impact the yields of various crops, particularly in the northwest region of Bangladesh^11^. Pre-kharif (mid-March to mid-May) and Rabi (mid-November to mid-March) crops are highly susceptible to drought^12^. Every year, droughts of varying intensities have caused damage to around 2.32 million hectares of land^9^. In addition to agricultural loss, drought has social and environmental consequences such as loss of livelihoods, migration, food price hikes, loss of biodiversity, disease, land degradation, and so many others^1,13^. Hence, drought prediction studies are necessary to reduce the adverse impacts of drought events on water resources, agriculture, energy production, ecosystems, public safety, and the economy. They are critical for sustainable resource management and preparedness in the face of a changing climate^14^.

Climate change adaptation and coping strategies have remained a global concern for decades. One of the key reasons for the failure of disaster risk management in climate-vulnerable countries like Bangladesh is that the government always emphasizes response and recovery over monitoring, preparedness, and mitigation. In light of this, accurate drought projections are crucial for the sustainable management of agricultural resources. The erratic and spatial nature of drought, with varying intensity and frequency^10^, necessitates identifying rapid, consistent, and precise prediction models to quantify drought-related risks.

Several drought indices have been established in recent decades to monitor drought on regional and global scales^15–21^. Among them, the standardized precipitation index (SPI)^20–29^, standardized precipitation evapotranspiration index (SPEI)^15,18–21,27,28,30–38^, and Palmer drought severity index (PDSI)^39,40^ were widely used. Recently developed SPEI^15^ has the advantage of determining many types of drought^41^. The SPEI takes into account both the multi-scalar properties and straightforward computation of the SPI and the PDSI's sensitivity to shifts in evaporation demand; hence, broadly acceptable for monitoring and analyzing drought characteristics^42^.

Sustainable water management requires a reliable data-driven drought prediction model^43,44^. Traditional stochastic techniques, such as the autoregressive integrated moving average (ARIMA) and seasonal autoregressive moving average (SARIMA) models, were the most widely used for predicting droughts^45,46^. Recent applications of machine learning (ML) models offer the advantage of being more adaptable and robust for drought prediction^44,47–52^. ML models better capture complicated relationships between variables, handling nonlinearity and temporal dependencies. Additionally, ML models can be easily updated with new data, making them suitable for dynamically changing environments^53,54^. Several ML models, such as artificial neural networks (ANN), Fuzzy Logic (FL), support vector regression (SVR), random forests (RF), relevance vector machine (RVM), genetic programming (GP), and extreme learning machine (ELM) have been used in complex modeling interactions^46,55–57^. However, because of regional variability, no generalized or ideal model is acceptable for all climates situations^13^; rather, there is a risk of misleading model development^10,58^.

In Bangladesh, very little research has been done using ML methods^13,59,60^. All research was one or two region-specific^61^, and the development of ML models for drought forecasting on a more disaggregate regional scale has yet to unfold. Besides, researchers did not identify the relative importance of climatic attributes for drought assessment. The novelty of this study is that it fills these gaps by developing the best ML models for SPEI forecasting at multiple time scales and drought intensity mapping for Bangladesh. Specifically, the current study predicts SPEIs for 35 meteorological stations using 24 ML models. Then the deployed models' performance was evaluated to select the best drought forecasting features, and finally the spatio-temporal pattern of seasonal drought intensity and frequency was estimated for meteorological research stations across Bangladesh.

The weather patterns in Bangladesh are undergoing significant transformations due to its proximity to the equator and the rising global temperatures^62,63^. As a consequence of these changes, the country has been confronted with severe weather fluctuations, including frequent flooding and other calamities. In regions of Bangladesh where drought is a persistent concern, experts have observed an increase in the occurrence of droughts, attributable to alterations in temperature and precipitation patterns^64^. The impact of climate change is anticipated to result in more substantial economic losses from droughts^65^, affecting water resources and contributing to water scarcity^66^. These adverse consequences underscore the need for the development of robust forecasting and monitoring models for drought, enabling the timely formulation of strategies to manage drought-related risks^67^.

Effective drought forecasting is an indispensable component of drought management. Inadequate forecasting can lead to suboptimal management practices and potential harm to the environment. Consequently, there is a pressing demand for rapid, reliable, and accurate models for drought prediction that can furnish quantitative insights into impending drought-related threats. These models leverage the appropriate combination of input variables or drought indices to deliver precise drought forecasts^68^.

By exploring the interplay between climatic variables and machine learning models^69^, we aim to uncover the optimal combination that can provide actionable insights and early warning systems for mitigating the far-reaching impacts of droughts in the region. This research represents a vital step towards harnessing technology and data-driven approaches to address the pressing challenges posed by seasonal droughts, offering the potential to safeguard agricultural practices in Bangladesh. Our research holds significant implications for science, policy, and practice. Scientifically, it showcases the efficacy of machine learning methods in drought prediction and underscores the significance of integrating weather parameters in drought analysis. From a policy perspective, the study underscores the need for enhanced collaboration among stakeholders, including policymakers, researchers, communities, and local actors, to formulate effective adaptation strategies. In practical terms, the findings highlight the crucial importance of heightened weather condition monitoring in Bangladesh to mitigate the adverse effects of droughts on crop production and livelihoods.

The article is organized as follows: "Materials and methods" provides information on the study area, the utilized data, and the empirical settings. "Results" presents the study findings. A detailed discussion of the results is found in "Discussion", with a summary of conclusions and recommendations in "Conclusions and policy recommendations".

## Materials and methods

### Study area

**Figure 1:**
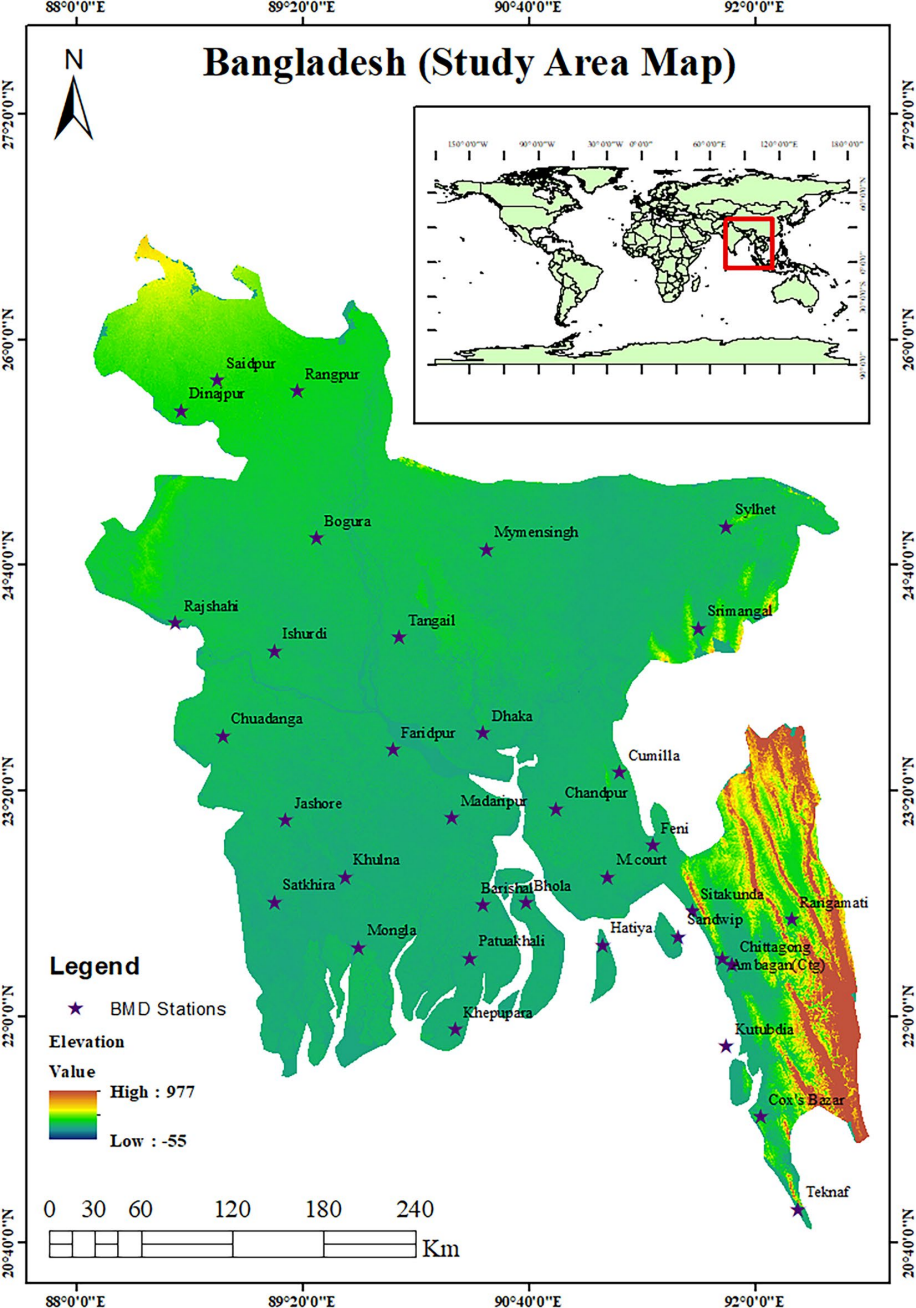
A map illustrating the locations of the examined meteorological stations in Bangladesh. The authors used ArcGIS 10.8 (https://www.arcgis.com/index.html) to generate the map, employing the administrative shapefile of Bangladesh in the process. Shapefile republished from the Bangladesh Agricultural Research Council (BARC) database (http://maps.barcapps.gov.bd/index.php) under a CC BY license, with permission from Computer and GIS unit, BARC, original copyright 2014.

Bangladesh, located in South Asia, stands out for its distinctive geographical and environmental features. Situated between latitudes 20°34' and 26°38' N and longitudes 88°01' and 92°41' E (Fig. 1), it shares borders with India to the west, north, and east, and Myanmar (Burma) to the southeast^70^. To the south, the Bay of Bengal forms a natural boundary. This densely populated nation boasts a complex landscape with expansive riverine systems, fertile alluvial plains, and the renowned Sundarbans, the world's largest river delta. Its predominantly low-lying terrain renders it susceptible to flooding, storm surges, and monsoon rains, with intermittent droughts affecting certain regions. Featuring a tropical climate, the country experiences average temperatures ranging from 12.8 to 31.1 °C. The annual rainfall varies from 1700 mm in the northwest to over 5000 mm in the southeastern region^13^, establishing Bangladesh as one of the world's wettest countries. The climate features distinct wet and dry seasons, profoundly impacting agriculture, the economy, and the predominantly agrarian livelihoods of its people. Bangladesh's unique geographical and climatic conditions have made it a focal point for research in areas such as climate change, agriculture, water resource management, and disaster preparedness, underlining its critical importance due to its vulnerability to environmental challenges and the potential for innovative solutions to enhance the well-being of its population.

### Data use

Climate records at the daily timescale from 35 meteorological stations were collected by the Bangladesh Meteorological Department (BMD) over the past 40 years, from 1981 to 2020 (Fig. 1). The climate variables were daily rainfall amount (mm), maximum temperature (°C), minimum temperature (°C), mean temperature (°C), sunshine hours (h), wind speed (ms^−1^), and relative humidity (%). In addition, potential evapotranspiration (PET) at the monthly timescale was calculated from the aforementioned climate variables. The Food and Agricultural Organization (FAO) recommends the Penman–Monteith (PM) equation^71^ as the single standard technique for calculating reference evapotranspiration (ET_0_), and it has been effectively utilized in Bangladesh. It integrates physiological and meteorological attributes and has been widely used around the world because of its intrinsic rationality and reliability^72^. Hence, the PM equation based on the weather parameters was utilized to compute the monthly ET_0_ over the research locations.

### Model selection process for data analysis

**Figure 2:**
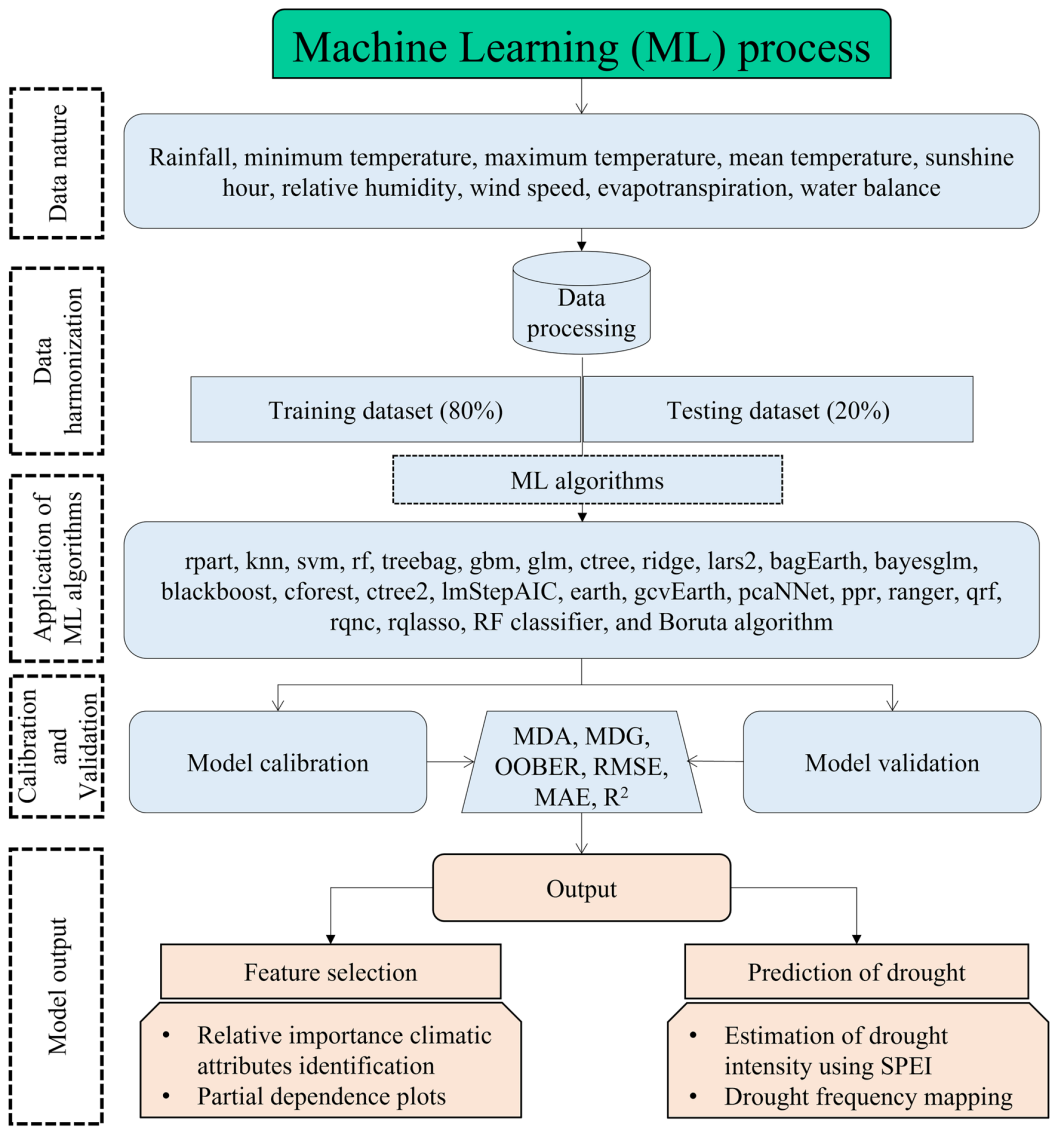
Conceptual framework of prediction of SPEI by ML algorithms for the study.

In this research, twenty-four (24) machine learning models were constructed to predict the Standardized Precipitation Evapotranspiration Index (SPEI) in various timescales, including 1-, 3-, 6-, and 12-month periods. The methodology, as illustrated in Fig. 2, encompassed the subsequent procedural phases:

- Data collection and preprocessing: The SPEI database was generated by utilizing meteorological variables from the SPEI computation model.
- Feature selection: The optimal features for classifying drought conditions were identified through the application of correlation and random forest classifier methods.
- Model selection and cross-validation: To implement machine learning algorithms, the dataset was randomly divided into training (80%) and testing (20%) sets, and all data points from each research station were imported into the R programming environment. Machine learning algorithms were employed on the training datasets, the models were validated using the testing datasets, and SPEI predictions were generated for various time scales. Each meteorological station's resulting output values were ranked to determine the most suitable machine learning models.
- Model evaluation: Models were evaluated using appropriate performance metrics, including RMSE, MAE, and R^2^.
- Model comparison: We compare the performance of different models and select the one that best aligns with our research objectives and provides the most accurate results.
- Validation and robustness testing: The selected model was further validated using different subsets of the data to assess its robustness.
- Model output and visualization: We focus on the presentation and interpretation of the model output through a robust visualization process.

#### Standardized precipitation evapotranspiration index (SPEI)

SPEI is a widely used technique for measuring drought dynamics over multiple time frames^15^. The SPEI is derived from precipitation and temperature data using a simple water balance to measure the effects of surface evaporation caused by increasing global temperatures^73^. It has an advantage over the SPI, because it combines rainfall and temperature into its computations, whereas SPI only uses rainfall^22^. The calculation of SPEI is based on the original SPI calculation procedure and hence uses the same index categorization criteria^15^.

The initial step in computing the SPEI is to determine the monthly water balance (D_i_), which is the difference between the precipitation (P_i_) and potential evapotranspiration (PET_i_). Afterwards, these values are combined at the desired timescales^74^ as:

1 $${D}_{i}={P}_{i}-{PET}_{i}$$

2 $${D}_{n}^{k}=\sum_{i=0}^{k-1}\left({P}_{n-i}-{PET}_{n-i}\right), n\ge k$$

where, k = 1, 3, 6, and 12 for SPEI = 1, 3, 6, and 12 is the aggregation timescale, and n is the nth month. Using the log-logistic probability distribution, the D-series was fitted. The cumulative distribution function F(x)^75^ can be expressed as follows:

3 $$F \left(x\right)={\left[1+{\left(\frac{\alpha }{x-\gamma }\right)}^{\beta }\right]}^{-1} k$$

where, the parameters of scale, shape, and location are written as α, β, and γ, respectively. The SPEI value is derived from the standard value provided:

4 $$SPEI=W-\frac{{c}_{0}+{c}_{1}W+{c}_{2}{W}^{2}}{1+{d}_{1}W+{d}_{2}{W}^{2}+{d}_{3}{W}^{3}}$$

5 $$W=\sqrt{-2{\text{ln}}(P)} for P\le 0.5$$

where P indicates the likelihood of exceeding a certain D value, and F(D) represents the cumulative distribution of D. When P is greater than 0.5, it is replaced with the non-exceed likelihood (F(D) = 1 − P) and the direction of the derived SPEI is inverted. The constants are c_0_ = 2.5155, c_1_ = 0.8028, c_2_ = 0.0103, d_1_ = 1.4328, d_2_ = 0.1892 and d_3_ = 0.0013^43^.

In this study, we estimated SPEI at time scales of one month (SPEI1), three months (SPEI3), six months (SPEI6), and a year (SPEI12). These estimates were used to measure the impact of precipitation deficits in the short term on agricultural drought. According to the SPEI classification criteria, the value of SPEI ≥ 0 indicates no drought, − 1.0 < SPEI < 0 indicates mild drought, − 1.5 < SPEI ≤  − 1.0 indicates moderate drought, − 2.0 < SPEI ≤  − 1.5 indicates severe drought and SPEI ≤  − 2.0 indicates extreme drought^76^. The greater the value of the SPEI in the negative, the more severe the drought.

We also estimated the severity of drought. A drought event's duration (m) equals the number of months between its start (included) and end month (not included). The absolute value of the total of all SPEI values during a drought event is known as severity (S_e_). A drought event's intensity (DI_e_) is defined as severity divided by duration^77^. The greater the DI_e_ number, the more severe the drought. The formulae are as follows:

6 $${S}_{e}={\left|\sum_{j=1}^{m}{Index}_{j}\right|}_{e}$$

7 $${DI}_{e}=\frac{{S}_{e}}{m}$$

where, e, j, Index_j_, m, S_e_, and DI_e_ are the drought event, month, SPEI value in month j, duration, severity, and intensity of a drought event e, respectively.

#### Best feature selection criteria

Feature selection is a widely used process of selecting the best features that can significantly influence the predicted outcomes, increasing model performance and accuracy, and reducing running time^78,79^. We considered two different feature selection approaches, random forest (RF), and the Boruta algorithm, to identify the most significant weather variables that affect SPEIs. The Boruta and *Caret* packages were used for feature selection in RStudio software. A brief overview of these two techniques is provided here:

##### Random forest

RF employed Mean Decrease Accuracy (MDA) and Mean Decrease Gini (MDG) to select variables^80^. When a variable is left out of the model, the MDA value represents how much precision is compromised. The more accuracy lost the more importance of the variable for successful classification. The MDG measures the contribution of each variable to the homogeneity of the random forest's nodes and leaves. The greater the MDG score, the greater the significance of the variable in the model^81^. Additionally, we used dichotomy method as a rapid variable screening technique. Time series of SPEI and other related weather variables were used to find a proficient and robust estimation of the best classifier. As a rule, the majority of scholars employed permuting out-of-bag (OOB henceforth) error or impurity to evaluate the significance of a single variable^82–84^, whereas we employed both. The OOB error is a bootstrap aggregation-based approach for assessing the prediction error of random forests, boosted decision trees, and other machine learning models^85^.

##### Boruta algorithm

The Boruta algorithm was introduced by Kursa and Rudnicki^86^. This technique attempts to reduce misleading outcomes due to correlations and random fluctuations by introducing more randomization and collecting results from the entire set of randomized samples. The relative importance of the climatic variables was identified for the estimate approach by following the steps outlined in Ebrahimi-Khusfi^87^, and Kursa and Rudnicki^86^.

#### Machine learning algorithms

We considered analyzing 24 distinct machine learning algorithms from various ML fields to determine the correlation between drought prediction and the weather attributes. The prediction of multiscale SPEI1, SPEI3, SPEI6, and SPEI12 considered tree-based algorithms, regression, and classification models. We used multiple predictive modeling techniques employing a variable selection algorithm. These methods included linear least squares models and penalized linear, additive, and recursive partitioning models, all implemented with R programming code for variable selection and prediction (Table 1).

**Table 1:** List of 24 machine learning methods and their characterizations.

Sl. no	Method	Description	Type	R packages	Reference
1	rpart	CART	Regression, classification	Rpart	Breiman and Freedman^122^, Breiman and Ihaka^123^, Therneau et al.^124^
2	knn	k-nearest neighbors	Regression, classification	Kknn	Uddin et al.^125^
3	svm	Support vector machines with radial basis function kernel	Regression, classification	Kernlab	Noble^126^
4	rf	Random forest	Regression, classification	randomForest	Breiman^80^
5	treebag	Bagged CART	Regression, classification	ipred, plyr, e1071	Breiman^80^, Kober et al.^127^
6	gbm	Stochastic gradient boosting	Regression, classification	gbm, plyr	Freedman^128^, Guelman^129^, Ridgeway^130^
7	glm	Generalized linear model	Regression, classification	MASS	Annette J. Dobson^131^
8	ctree	Conditional inference tree	Regression, classification	Party	Hothorn et al.^132^
9	ridge	Ridge regression	Regression	Elasticnet	Seegrist^133^, Zou and Hastie ^134^
10	lars2	Least angle regression	Regression	Lars	Efron et al.^135^
11	bagEarth	Bagged MARS	Regression, classification	Earth	Max Kuhn et al.^88^
12	bayesglm	Bayesian generalized linear model	Regression, classification	Arm	Dey et al.^136^
13	blackboost	Boosted tree	Regression, classification	Party, mboost, plyr, partykit	Chen^137^
14	cforest	Conditional inference random forest	Regression, classification	Party	Levshina^138^
15	ctree2	Conditional inference tree	Regression, classification	Party	Hothorn et al.^132^, Sarda-Espinosa et al.^139^
16	lmStepAIC	Linear regression with stepwise selection	Regression	MASS	Olusegun et al.^140^
17	earth	Multivariate adaptive regression spline (MARS)	Regression, classification	Earth	Friedman and Roosen^141^, Milborrow et al.^142^
18	gcvEarth	MARS generalized cross validation (GCV) penalty per knot	Regression, classification	Earth	Milborrow et al.^142^
19	pcaNNet	Neural networks with a principal component step	Regression, Classification	Nnet	Ripley^143^
20	ppr	Projection pursuit regression	Regression	Stats	Farikha et al.^144^
21	ranger	Random forest	Regression, classification	e1071, ranger, dplyr	Wright et al.^145^
22	qrf	Quantile random forest	Regression	quantregForest	Li and Peng^146^
23	rqnc	Non-convex penalized quantile regression	Regression	rqPen	Bello et al.^147^, Ma et al.^148^
24	rqlasso	Quantile regression with LASSO penalty	Regression	rqPen	Ciner et al.^149^

The study measured the performance of each ML algorithm independently. ML algorithms employ various statistical, probabilistic, and optimization methods to extract useful patterns from large and complex datasets that are unstructured and derived from past experiences^61^. Time series data is characterized by a sequential order, where each observation is influenced by the preceding observations. Applying traditional cross-validation techniques to time series data can introduce a significant source of bias since it violates the temporal structure of the data. To obtain robust and reliable estimates of a model's performance, assess its generalization capabilities, and make informed decisions in model selection, using tenfold cross-validation with five repetitions, the algorithms were trained and compared. It helps reduce the impact of random variations, provides more stable performance metrics, and aligns with established practices in the field of machine learning and data analysis. All predictive models were trained utilizing the *Caret* package's interface for the train function^88^. The train function generates the parameter tuning by determining the values that maximize root-mean-square error (RMSE) accuracy. The data were divided into training (80%) and test (20%) sets. The ML function determines the optimal subset of predictors for the best accurate model. Finally, the study rated lists of predictors from each training approach for the final models.

#### Model evaluation metrics

Model validation is a necessary step of ML modeling for evaluating the accuracy and reliability of models. Scholars employed various statistical metrics for this purpose^89–91^. We used RMSE, MAE, and R^2^ to evaluate the performance of the constructed models. The statistical evaluation metrics are the following for all parameters:

8 $$RMSE=\sqrt{\frac{\sum_{i=1}^{N}{\left({Y}_{obs}-{Y}_{pred}\right)}^{2}}{N}}$$

9 $$MAE=\frac{\sum_{i=1}^{N}\left|{Y}_{obs}-{Y}_{pred}\right|}{N}$$

10 $${R}^{2}=\frac{\sum_{i=1}^{N}\left({Y}_{obs}-{\overline{Y} }_{obs}\right)\left({Y}_{pred}-{\overline{Y} }_{pred}\right)}{\sqrt{\sum_{i=1}^{N}{\left({Y}_{obs}-{\overline{Y} }_{obs}\right)}^{2}}\sqrt{\sum_{i=1}^{N}{\left({Y}_{pred}-{\overline{Y} }_{pred}\right)}^{2}}}$$

where, *Y*_*obs*_ and *Y*_*pred*_ indicates the actual and predicted dependent variable, respectively, with N denoting the number of observations. As a general rule, models with a lower RMSE, MAE, and a larger R^2^ during testing were deemed more accurate for accuracy of good prediction model.

#### Partial dependence plots (PDPs) method

The model-independent method is based on determining the "flatness" of the PDPs of each feature. PDPs assist in visualizing the influence of low cardinality feature space subsets on the estimated prediction surface, such as main effects and two/three-ways interaction effects. The PDPs provides model-independent interpretations and can be developed via a supervised machine learning approach. We train a projection pursuit regression (PPR) model and use the pdp package to generate PDPs for each feature^92^. The PDPs can be misled in the presence of substantial interactions^93^. To solve this issue, Goldstein et al.^93^ developed individual conditional expectation (ICE) charts, which are available in the R programming package ‘ICEbox’. The ICE plots display the estimated association between the response and a predictor of relevance for each observation. Consequently, the PDP for an activity predictor may be computed by averaging the proper ICE curves' overall data.

## Results

### Correlation analysis

**Figure 3:**
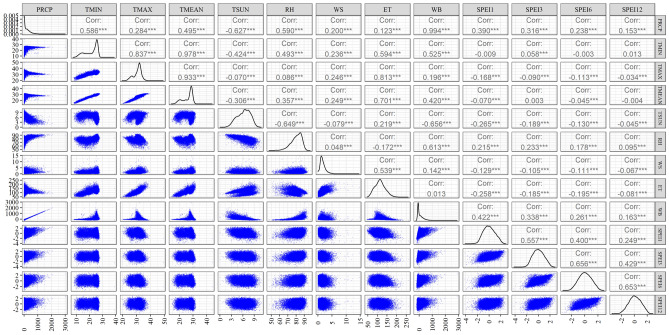
Correlation coefficients among the weather parameters and SPEI’s values.

In this study, the meteorological indices, standardized precipitation evapotranspiration index at multiple time scales (SPEI1, SPEI3, SPEI6, and SPEI12), were chosen to assess the drought conditions of Bangladesh. The study aimed to explore their associations with nine distinct weather parameters: precipitation (PRCP), minimum temperature (TMIN), maximum temperature (TMAX), average temperature (TMEAN), total sunshine (TSUN), relative humidity (RH), wind speed (WS), evapotranspiration (ET), and water balance (WB) (Fig. 3). Regarding SPEI1, the correlation coefficients revealed statistically significant (p < 0.05) and positive associations with rainfall (0.390), relative humidity (0.215), and water balance (0.422). Conversely, there were statistically significant negative correlations with maximum temperature (−0.168), mean temperature (−0.070), total sunshine hour (−0.265), wind speed (−0.129), and evapotranspiration (−0.258). The correlation results revealed that meteorological indices at the 3-month time scale (SPEI3) exhibited a nearly identical relationship like SPEI1 with meteorological variables. Specifically, rainfall, minimum temperature, relative humidity, and water balance displayed significant positive associations with SPEI3, with correlation coefficients of 0.316, 0.058, 0.233, and 0.338, respectively. On the other hand, there were statistically significant negative correlations with maximum temperature (−0.090), total sunshine hour (−0.189), wind speed (−0.105), and evapotranspiration (−0.185).

Also, for both SPEI6 and SPEI12, the correlation analysis reveals significant (p < 0.05) positive associations with rainfall (0.238 and 0.153), relative humidity (0.178 and 0.095), and water balance (0.261 and 0.163) (Fig. 3). In the case of SPEI6 and SPEI12, it's noteworthy that a negative and statistically significant relationship was observed with certain meteorological parameters. Specifically, maximum temperature exhibited negative correlations of −0.113 for SPEI6 and −0.034 for SPEI12. Likewise, total sunshine hour displayed negative correlations of −0.130 for SPEI6 and −0.045 for SPEI12. Additionally, wind speed showed negative correlations of −0.111 for SPEI6 and −0.067 for SPEI12. Furthermore, evapotranspiration had particularly significant negative correlations, with values of −0.195 for SPEI6 and −0.081 for SPEI12. These results indicate that as SPEI6 and SPEI12 values decreased, these meteorological parameters tended to increase, and the relationships were statistically significant.

### Identification of best climatic attributes for different SPEIs

**Figure 4:**
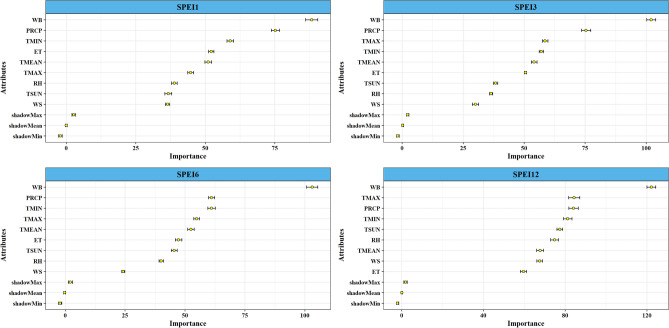
Best feature combination of predictor variables based on the Boruta algorithm.

Based on the results depicted in Fig. 4, the critical variables for the SPEI1 time scale were identified as WB, PRCP, TMIN, and ET. For the SPEI3, SPEI6, and SPEI12 time scales, the most significant variables were WB, PRCP, TMAX, and TMIN. Consequently, WB, PRCP, TMAX, and TMIN emerged as the predominant factors influencing the construction of drought prediction models using machine learning.

The random forest classifier algorithms tuned using cross-validation ten folds and five repeats were summarized, and the performance of the RF classifier was presented in Table 2. The best three contributors for the SPEI1 model were WB, PRCP, and TMIN with the highest percentage values, and the overall OOB error rate for SPEI1 model was 17.77%. However, the worse contributor was identified as WS, RH, and TSUN, getting the lowest percentage among the variable for SPEI1. We found that the SPEI3 model had the same contributors as the SPEI1 model. The best predictor for SPEI6 was WB, followed by TMIN, PRCP, and TMAX. The worse contributor was WS, which had the lowest percentage value of MDA and MDG, but the OOB error rate was 23.05%. The annual time scale (SPEI12) also has a vital role in identifying and predicting drought. The best and most significant contributors for SPEI12 were WB, TMIN, and TMAX, and the OOB error rate was low at 6.59%. Thus, the findings indicated that WB, PRCP, TMAX, and TMIN were the most significant contributors to drought model prediction across different time scales of Bangladesh.

**Table 2:** Performance of RF classifier model with different SPEI time scales derived from different feature combinations.

Attributes	SPEI1			SPEI3			SPEI6			SPEI12		
	MDA (%)	MDG (%)	OOB error rate (%)	MDA (%)	MDG (%)	OOB error rate (%)	MDA (%)	MDG (%)	OOB error rate (%)	MDA (%)	MDG (%)	OOB error rate (%)
PRCP	13.65	15.24	17.77	13.76	15.63	19.19	10.84	14.44	23.05	9.90	17.02	6.59
TMIN	12.67	10.85		11.90	10.32		12.58	11.70		13.40	9.19	
TMAX	9.15	9.10		11.46	9.62		10.75	9.70		12.97	9.55	
TMEAN	10.03	9.64		10.72	9.43		10.39	10.13		9.33	6.91	
TSUN	7.53	7.35		7.38	7.80		10.00	8.80		10.61	8.42	
RH	8.86	6.88		7.57	8.09		8.75	9.43		10.07	10.45	
WS	9.01	6.29		7.13	6.57		5.98	7.39		9.44	7.56	
ET	11.04	9.58		10.28	8.50		9.76	8.71		7.36	8.72	
WB	18.06	25.07		19.81	24.03		20.94	19.70		16.91	22.18	

*MDA* mean decrease accuracy, *MDG* mean decrease Gini, *OOB* out-of-bag estimate of error rate.

### Performance evaluation of ML models for different SPEIs during the training phase

To assess the precision and performance of the model, we utilized metrics including mean absolute error (MAE), root mean square error (RMSE), and the coefficient of determination (R^2^). In our interpretation, a well-performing model is characterized by lower MAE and RMSE values and a higher R^2^ value. For predicting SPEI1 (Standardized precipitation evapotranspiration index at a 1-month time scale), the ranger model stood out as the most accurate, boasting an impressive R^2^ value of 0.689, indicating its substantial explanatory power (Fig. A1a). Additionally, it displayed relatively lower RMSE (0.547) and MAE (0.417) values, reflecting close alignment with actual data. The rf, svm, and cforest models also performed well, securing the second, third, and fourth positions, respectively, in SPEI1 prediction accuracy. Conversely, the CART and lars2 models exhibited lower accuracy in predicting SPEI1.

According to Fig. A1b, evaluating the performance of machine learning models for predicting the 3-month time scale drought (SPEI3), the ranger model emerged as the top performer with an R^2^ of 0.602, RSME = 0.600, and MAE = 0.454, indicating robust predictive capabilities. The rf model closely followed, achieving an R^2^ of 0.602, RSME = 0.601, and MAE = 0.456. The qrf and cforest models secured the third and fourth positions with R^2^ values of 0.598 and 0.593, respectively. In contrast, the lars2 and rqlasso models exhibited lower accuracy in predicting SPEI3. The analysis was extended to predict SPEI6, which represents a 6-month drought index. In this case, the ranger model continued to exhibit strong performance, with the highest R^2^ value of 0.512, and the lowest RSME (0.661) and MAE (0.522). The rf, svm, and cforest models closely followed. Once again, the CART and lars2 models were less accurate in predicting SPEI6 (Fig. A1c). For the 12-month time scale drought (SPEI12), the qrf model emerged as the top performer with the highest R^2^ value of 0.871, signifying its exceptional predictive accuracy of drought condition. The ranger, rf, and cforest models also demonstrated robust performance, while the CART and lars2 models exhibited comparatively lower accuracy in forecasting SPEI12 (Fig. A1d).

The Taylor diagram, a widely used pictorial tool, serves to evaluate the performance of ML models^13,83^. This diagram visualized the spatial pattern of calculated (reference field) against predicted (test field) multi-time scale SPEI values^94^. Fig. A2 depicts the Taylor's diagram, incorporating metrices such as RMSE, correlation coefficient, and standard deviation for SPEI1, SPEI3, SPEI6, and SPEI12. It was observed from the figure that all ML models exhibited a standard deviation of less than one across each SPEI time scale. Similarly, the correlation results varied from 0.40 to 0.80 for SPEI1, SPEI3, and SPEI6, while ranging from 0.40 to 0.95 for SPEI12. Additionally, centered RMSE was more scattered in SPEI12 compared to other time scales. Notably, the ranger model consistently outperformed than other models across all time scales.

### Observed and predicted performance of ML models for countrywide datasets

The scatter plot illustrating the performance of observed and predicted SPEIs is presented in Fig. A3a–d. For SPEI1 (Fig. A3a), actual vs predicted R^2^ values ranged from 0.26 to 0.69, with 46% (11 models) displaying R^2^ values equal to or exceeding 60%. Notably, models such as ranger, rf, qrf, and cforest demonstrated high accuracy during validation. In the case of SPEI3, 21% (5 models) exhibited an R^2^ value surpassing 0.60, with ranger, rf, qrf, cforest, and svm models showcasing exemplary prediction performance (Fig. A3b). Approximately 17% of SPEI6 models achieved an R^2^ value greater than 0.49, with ranger, svm, rf, and qrf models standing out (Fig. A3c). The R^2^ square values for SPEI12 ranged from 0.29 to 0.87, and during validation, qrf, ranger, and rf models exhibited R2 values exceeding 80%, indicating highly accurate prediction performance (Fig. A3d). In conclusion, the drought prediction performance of ML models varied across different time scales, with ranger, rf, and qrf models demonstrating consistency.

Another approach to identify the best model with fewer outlier issues is presented in Fig. A4a–d, providing an overview of the actual and predicted scenario of ML models, including outlier considerations. All models, across various time scales, showcased optimal performance when addressing the outlier problem. Empirical data supported the superiority of ranger, rf, and qrf models for precise drought prediction in Bangladesh. In contrast, CART, lars2, pcaNNet, ImStepAIC, and some other models struggled to overcome outlier values, negatively impacting their performance compared to the actual values of SPEI1, SPEI3, SPEI6, and SPEI12.

### Ranking the best predictive model for different regions at multiple timescales of SPEIs

**Figure 5:**
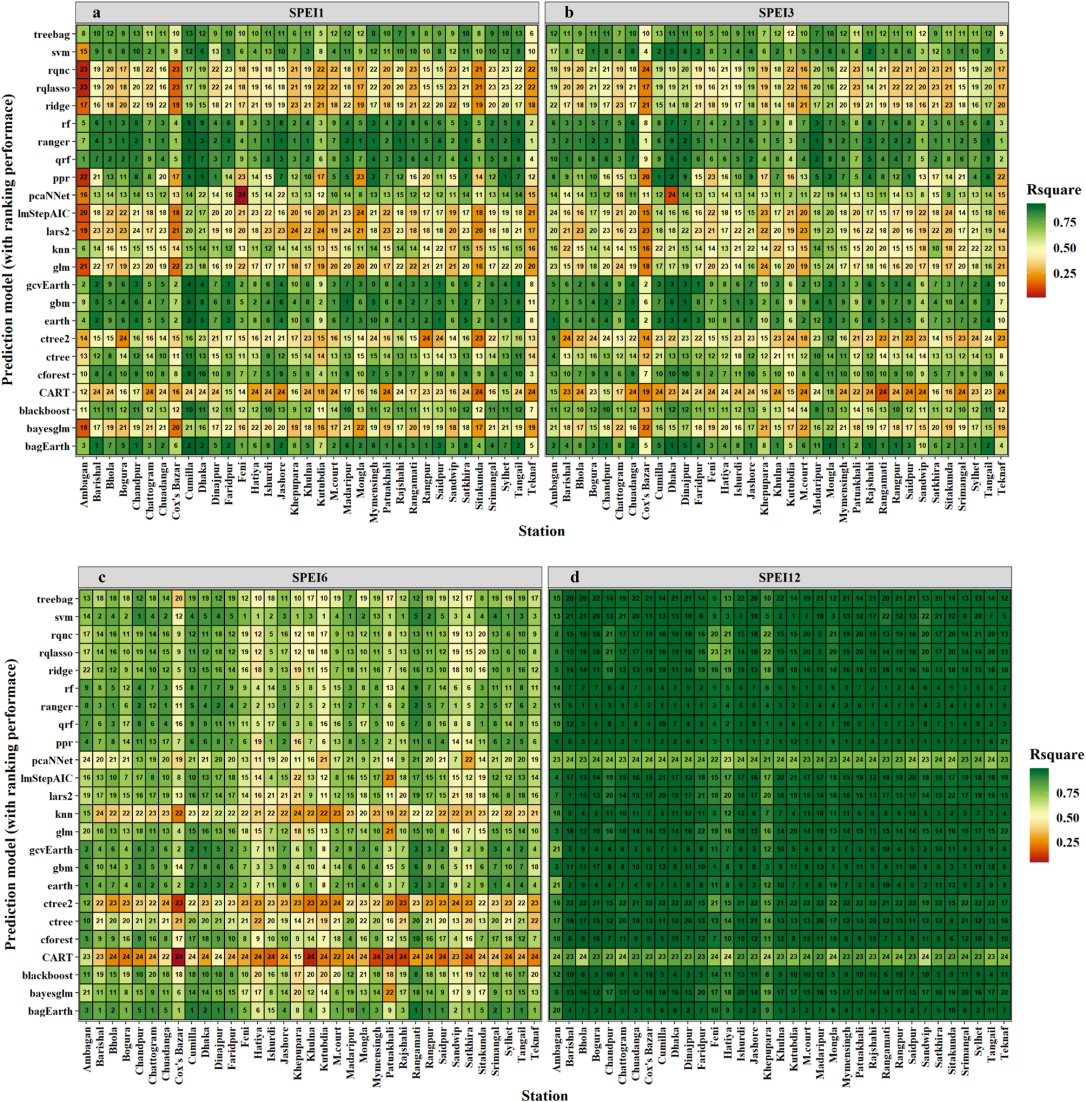
Heatmap illustrates the region-specific ML model selection for drought assessment based on R^2^, MAE, and RMSE values. Various colors indicate the strength of the R^2^ values. The region-specific ranking of ML models for predicting (**a**) SPEI1, (**b**) SPEI3, (**c**) SPEI6, and (**d**) SPEI12 was displayed by the added value label in the middle of the box. Greater R^2^ and lower MAE and RMSE values defined the performance ranking scale of the model.

**Figure 6:**
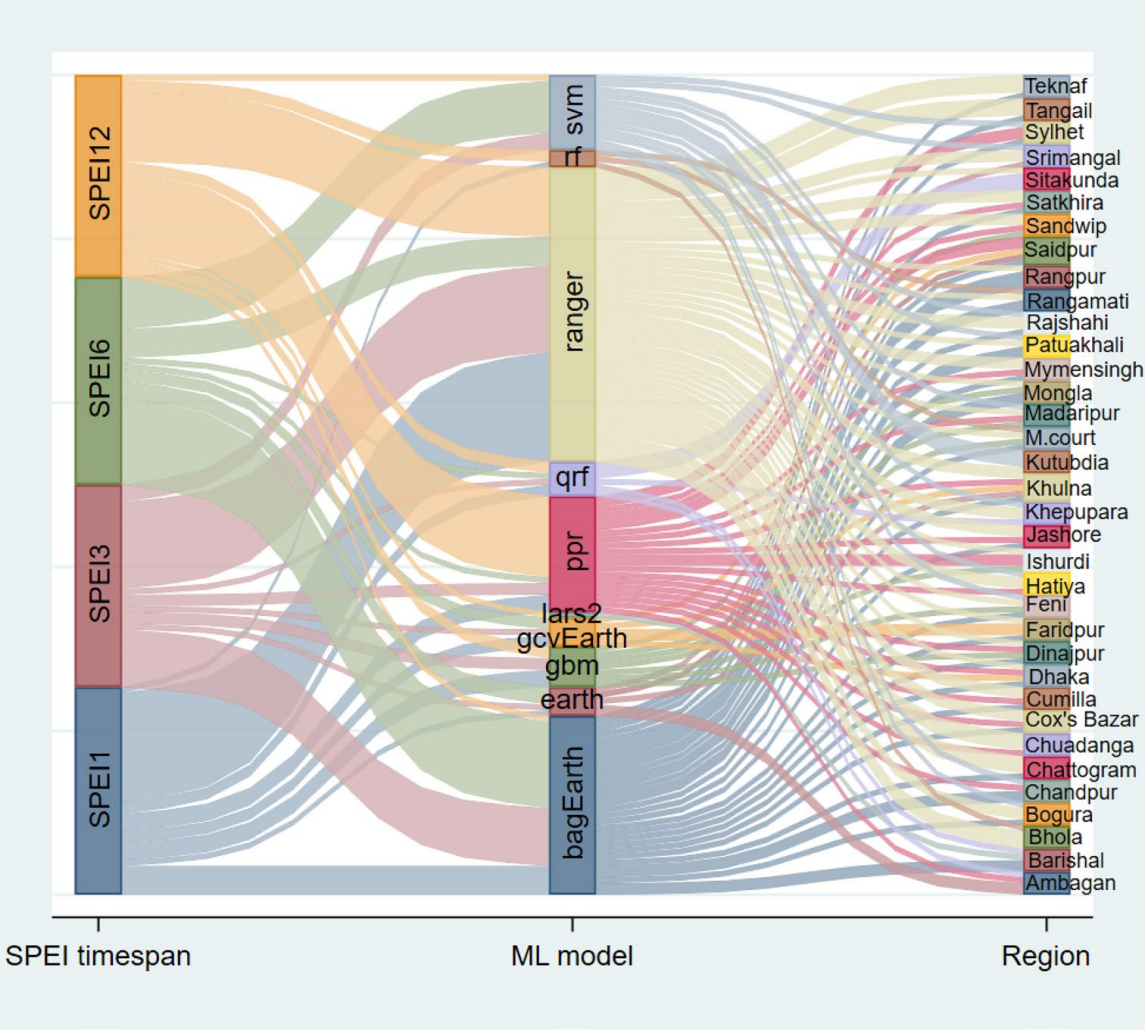
The Sankey graph illustrates a visualization of drought prediction models in Bangladesh across different timescales and regions.

Given the geographical positioning and climatic unpredictable changes across the country, a one-size-fits-all model would not be suitable for predicting drought in all locations. Here, we demonstrated a regional drought forecast for 35 meteorological stations using 24 ML models at various SPEI periods (Fig. 5a–d). The performance of each model was graded using higher R^2^ and lower MAE and RMSE values, illustrated through a heatmap. The results revealed that the best model differed across geographical locations and timespan. In the northern region of Bangladesh, for instance, at Rajshahi station in SPEI1 and SPEI12, the ranger model performed the best (ranked first). Similarly, the bagEarth and svm models had the highest performance in SPEI3 and SPEI6, respectively. In the southern region of Khulna, the ranger, bagEarth, earth, and ppr models performed most well at SPEI1, SPEI3, SPEI6, and SPEI12, respectively. Regarding regional representation, the ranger model demonstrated superior performance in 79% and 63% of regions (out of 35 stations) for SPEI1 and SPEI3, respectively. Conversely, for SPEI6 and SPEI12, the bagEarth and ppr models excelled, leading in 63% and 58% of regions, respectively (Fig. 6). However, the heatmaps of SPEI1 and SPEI3 were identical, and the usual R^2^ values for SPEI12 were fairly high. Refer to Figs. 5a–d and 6 for detailed information on the best model for each region.

### Evaluation of best predictive models for the specific region

**Figure 7:**
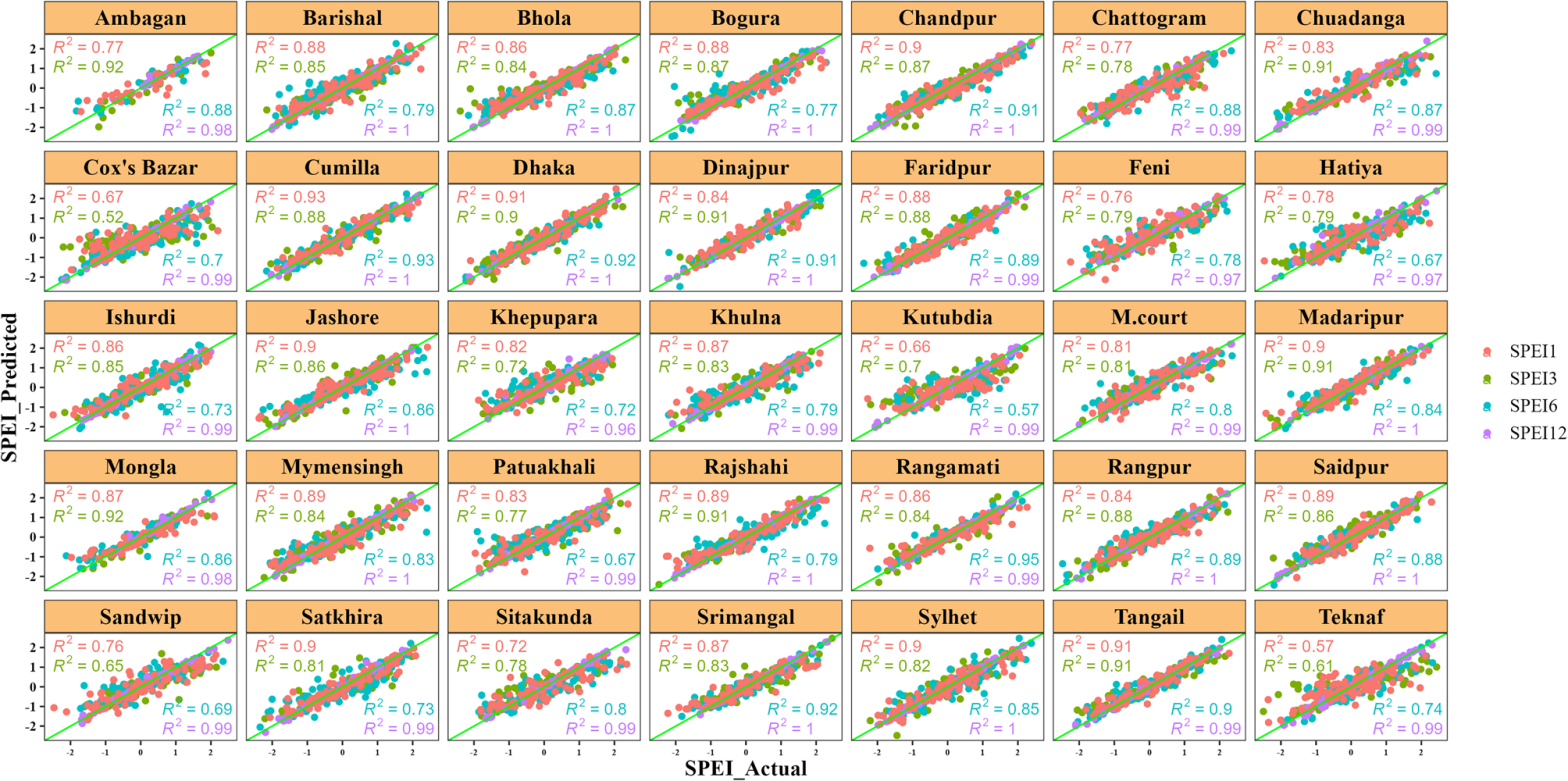
Region-specific best predictive ML models' performance.

We used scatter plots of fitted vs. observed values and R^2^ values to evaluate the best model for use across all regions and different time periods of SPEIs (Fig. 7). For SPEI1, the R^2^ values ranged from 0.57 to 0.93, indicating a positive correlation between the ML model and the observed data, with the model explaining 57–93 percent of the variance in the fitted data. Similarly, for SPEI3 and SPEI6, the R^2^ values ranged from 0.52 to 0.92 and 0.57 to 0.95 respectively, signifying a positive correlation between the ML models and the observed data, with the model’s explaining 52–92 percent and 57–95 percent of the variance in the fitted data, respectively. Lastly, the high R^2^ value for SPEI12 suggested a better fit for the model. Thus, confirming the validity of the models selected for drought prediction in Bangladesh across different time scales and regions.

**Figure 8:**
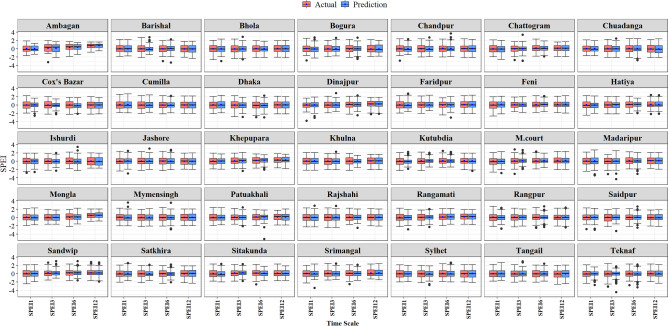
Box plot presentation of the best ML model performance of SPEI prediction for multiple time scales at the 35 investigated meteorological stations of Bangladesh.

To examine the accuracy in SPEI prediction, boxplots of 25%, 50%, and 75% quantile values for both observed and projected SPEI are shown in Fig. 8. The figure illustrates that the identified best models adequately simulated the variability in SPEI1, SPEI3, SPEI6, and SPEI12 values across different regions. While many predicted SPEI values exhibited minimum fluctuation, except the observed values displayed a wide range when SPEI fell below -2 or exceeded 2 in a few cases. However, the identified best model showed better accuracy in simulating the variability and quantile of SPEIs compared to others. All prediction models exhibited enhanced performance in modeling SPEI quantiles across various SPEI scales, particularly at higher orders.

### Partial dependence plot for fitted projection

**Figure 9:**
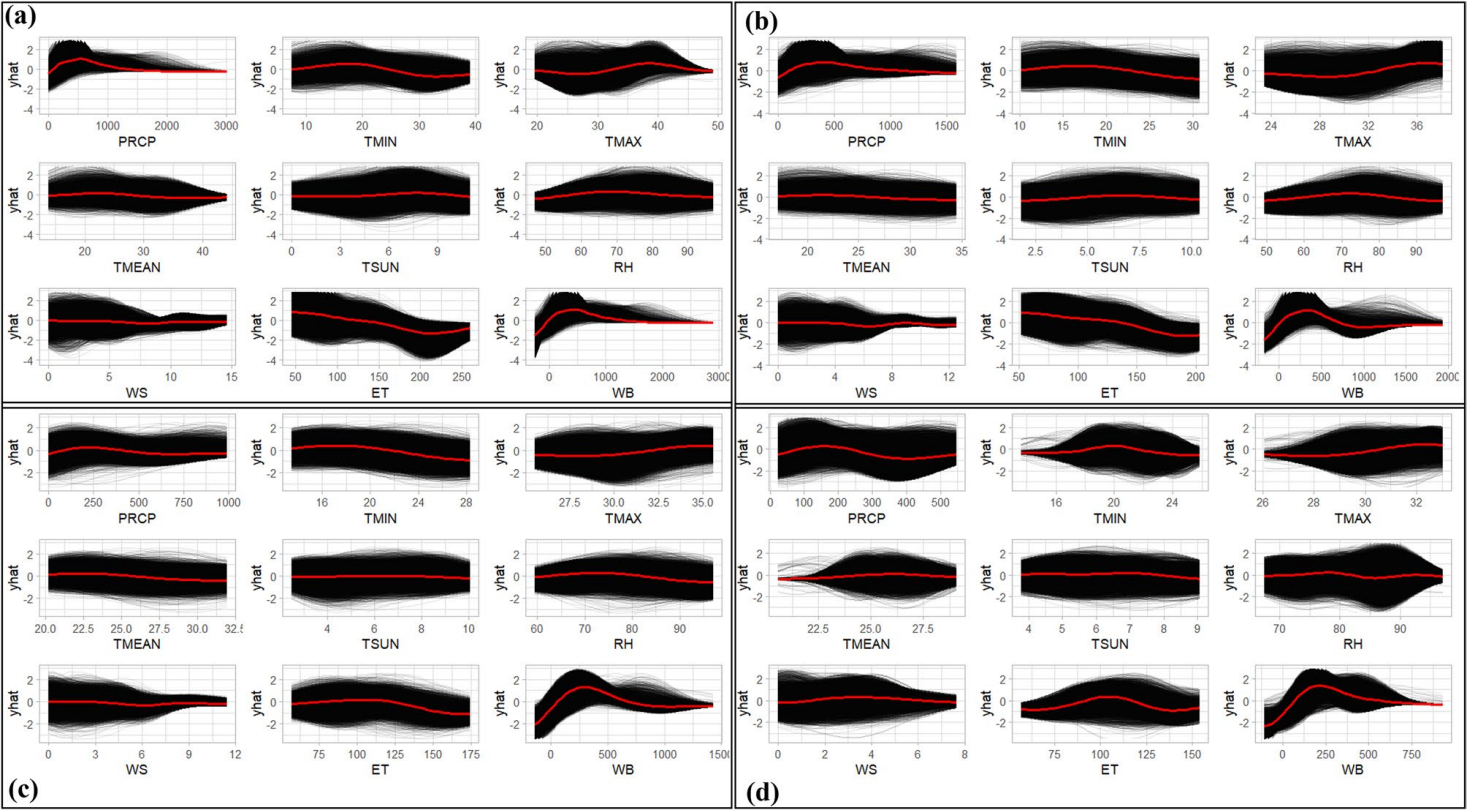
Fitted a partial dependence plot using the ICE curve method for each climatic feature against SPEI (yhat) for different time scales (**a**) SPEI1, (**b**) SPEI3, (**c**) SPEI6, and (**d**) SPEI12. Black and red curves denote ICE curves and their average value.

We employed a multivariate regression model to assess the significance of weather variables, and their relative effect on predicting SPEIs. In our analysis, ICE curves (depicted in black) and their mean (illustrated as the red line) were employed to visualize the relationships between individual weather attributes and the predicted SPEIs. This approach allowed us to identify critical climatic threshold values (Fig. 9). The findings revealed that Bangladesh experienced a range of drought moderate to severity levels, with a deficit of 92, 95, 115, and 143 mm of average rainfall over one, three, six, and twelve months, respectively. Temperature played a crucial role, with minimum, maximum, and mean temperatures exceeding 20.7 ± 1.1, 30.9 ± 0.7, and 25.9 ± 0.8 ℃, respectively, resulting in severe drought conditions across these time scales. Similarly, we observed that extended periods of sunshine hours and relative humidity surpassing 6.3 ± 0.6 h and 77.3 ± 1.3%, respectively, contributed to drought conditions. Low wind speeds below 1.9 ± 0.2 m/s and high evapotranspiration exceeding 123 ± 10 mm at all four-time scales also played a significant role in inducing drought in the country. Furthermore, the water balance was identified as a substantial factor affecting SPEI prediction. Below-average water balance levels, specifically 116, 143, 148, and 190 mm for one, three, six, and twelve months, respectively, were associated with drought occurrences in Bangladesh. These findings provide critical insights into the complex interplay of weather variables and their impact on drought patterns in the study region.

### Spatio-temporal pattern of seasonal drought intensity and frequency

**Figure 10:**
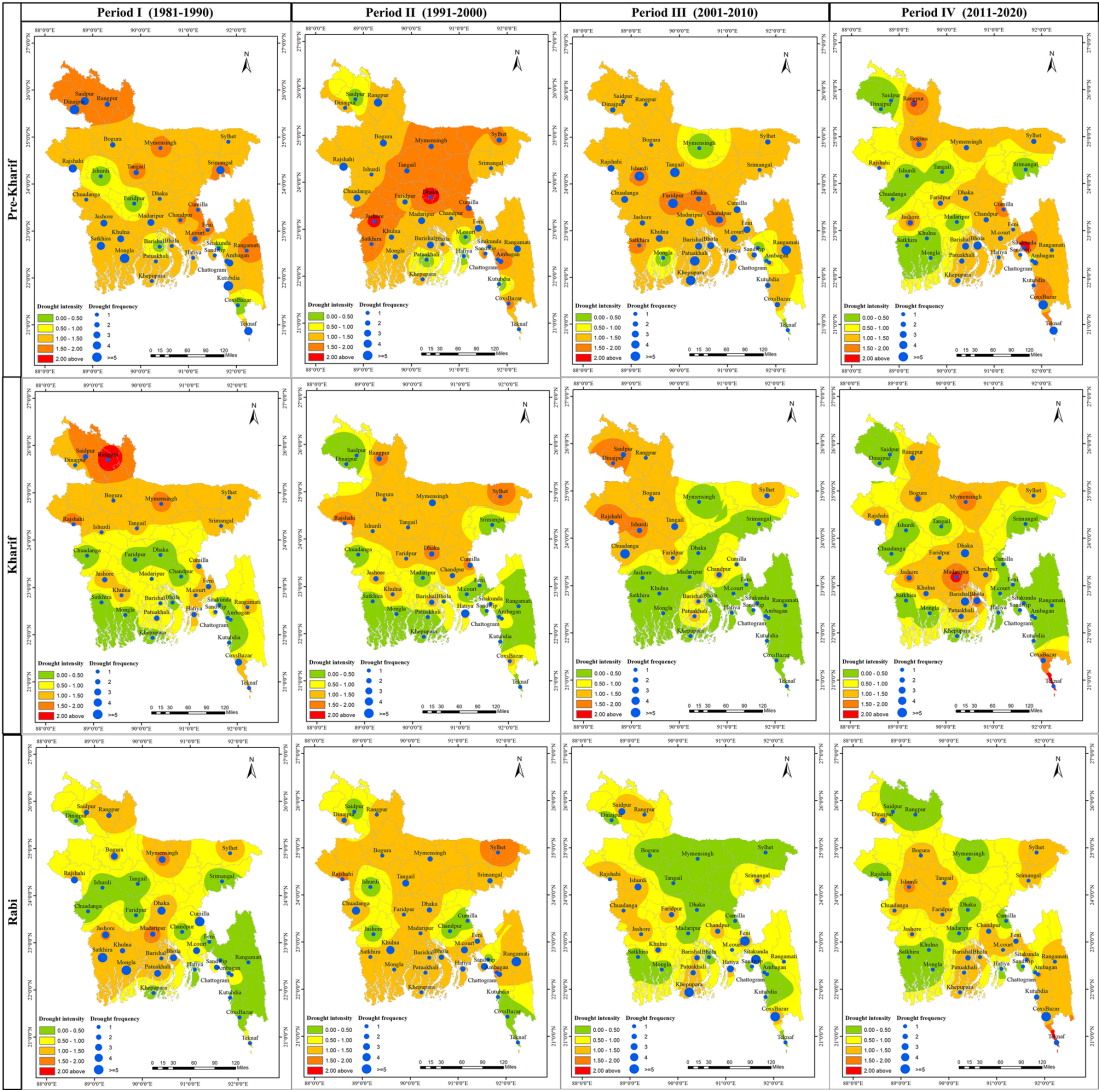
Spatio-temporal pattern of drought intensity and frequency based on the best predicted model for Pre-kharif, Kharif, and Rabi seasons over four decades (1981–1990, 1991–2000, 2001–2010, and 2011–2020) of Bangladesh. The authors used ArcGIS 10.8 (https://www.arcgis.com/index.html) to generate the map, employing the administrative shapefile of Bangladesh in the process. Shapefile republished from the Bangladesh Agricultural Research Council (BARC) database (http://maps.barcapps.gov.bd/index.php) under a CC BY license, with permission from Computer and GIS unit, BARC, original copyright 2014.

Using the region-specific best selected model based on SPEI influential meteorological parameters, we predicted the seasonal intensity and frequency of drought over time in Bangladesh (Fig. 10). We divided the forty years into four periods, i.e., Period I: 1981–1990, Period II: 1991–2000, Period III: 2001–2010, and Period IV: 2011–2020. Results showed that while drought intensity has decreased over time, but the return period has become more frequent. Spatially, the drought intensity shifted from the northern to central and southern zones of the country. In periodic assessment, the period with the most severe drought intensity was Period II. Notably, the frequency of drought has increased in Periods III and IV, indicating an increase in the number of droughts that occurred twice a decade in the past.

Season-wise, Pre-kharif was the most common season for drought compared to other seasons. During the Period I of the Pre-kharif season, the northern, eastern, and a few southern regions of the country were primarily affected by severe drought. During Period II, the northern and the majority of the central regions were most affected by drought, while the severity of drought in the north and center regions gradually relieved in Period III. In Period IV, the drought severity has been more prevalent in the Rangpur, Bogura, Sylhet, Mymensingh, Cumilla, Jashore, Sitakundu, Kutubdia and parts of southern regions in Bangladesh. Except for Rajshahi, the drought intensity of the Barind tract (located mainly in the northwestern part) was so unpredictable and has decreased significantly in recent decades. Noticeably, we found that the intensity of drought (> 1.0) in Chattogram division has been affected continuously over the last forty years. The highest frequency of drought was observed during Period III (2001–2010). Among all periods, the spatial patterns of drought frequency had changed, and high crop-intensive areas had become more vulnerable in the Pre-kharif season.

During the Kharif season (June-October), the incidence of drought was lower than in the Pre-Kharif season, but the pattern was comparable. In Period I of the Kharif season, the northern region of the country was hit by a severe drought, and the central half of the region was affected by a moderate drought. The intensity of the drought shifted from the north to the central region during the succeeding decade. During Period III, the majority of the northwest region again witnessed a severe drought, while the rest of the county was affected by a mild drought. In Period IV, the frequency of drought increased relative to previous periods, and its intensity rose in the north-eastern, central, and southern regions of Bangladesh.

During the Rabi season in Period I, the southwest and a portion of the northern region experienced drought intensity larger than one, and drought frequency greater than three times the average. However, nearly the entire country faced drought conditions in the succeeding decade. Throughout the country, the drought intensity has reduced, but frequency increased in Period III. Again, during Period IV, the divisions of Barishal, Chattogram, and Sylhet, as well as parts of the central regions, had droughts with intensities more than one. This suggests that drought conditions were similarly erratic throughout the Rabi season.

Overall, the intensity and frequency of drought in Bangladesh have exhibited erratic patterns over the past forty years, with certain periods witnessing more severe droughts than others. Factors such as average rainfall, temperature, sunshine hours, relative humidity, wind speed, and evapotranspiration have collectively influenced drought intensity, along with water balance deficits. These factors have significantly influenced SPEI predictions, rendering regions with high crop intensity more susceptible. This underscores the crucial importance of comprehending the impacts of drought on food production and livelihoods in the region.

## Discussion

Bangladesh experiences a predominantly tropical climate characterized by high temperatures and humidity, with droughts being more prevalent than other climate stressors in the country^13^. The occurrence, severity, and duration of droughts vary based on meteorological, hydrological, and agricultural factors^95^. In an effort to develop best ML-based models for predicting seasonal droughts in Bangladesh, this study utilized data from 35 meteorological stations at four distinct time scales. Applying 24 ML models based on the SPEI, we compared them with individual station predictions at different time intervals, contributing to the formulation of a spatio-temporal drought management program. In alignment with previous studies by Alamgir et al.^96^ and Yaseen et al.^13^, our SPEI1 results indicated a short-term rainfall deficit, SPEI3 and SPEI6 suggested agricultural drought, and SPEI12 reflected reductions in river flow and groundwater levels, signifying hydrological droughts in Bangladesh.

The use of ML models guaranteed the robustness of the drought prediction. We observed precipitation, maximum and minimum temperature, and water balance was the essential elements for predicting droughts in Bangladesh. Similar findings has been found by Rahman and Lateh^8^. In Pakistan, relative humidity, temperature, and wind speed were the most significant meteorological characteristics for accurately predicting drought^97^. Using ML models, Zhang et al.^46^ determined that temperature and precipitation, air pressure, wind speed, relative humidity, and duration of sunshine have a substantial effect on drought in China. So, it is evident that the relative importance of meteorological parameters on drought prediction varies among geo-climatic and geographical segments. Zhang et al.^46^ also stated that rf, ranger, qrf, bagEarth, and svm were the best methods for annual drought prediction. In the context of model stability assessment, the ranger model has the highest chance for 1–5 rating 90% of the time in SPEI1, SPEI3, and SPEI12 time scales. On another side, svm and bagEarth have 91% and 86% probability on the SPEI6 time scale, respectively. The svm-based models exhibited great temporal and geographical drought characterization in Pakistan^97^. Similarly, we observed that the performance and efficacy of the model varied based on the time frame and location of its application.

Our exemplary findings urged for the adoption of regional models instead of a single unified model, considering regional spatio-temporal heterogeneity. This conclusion is drawn from the analysis of influential meteorological variables on the SPEI depicted in Fig. 4, and the identification of region-specific best models shown in Fig. 6. These models indicate a spatio-temporal shift in both the intensity and frequency of seasonal drought patterns in Bangladesh, as illustrated in Fig. 10. Notably, the intensity of drought in climate hotspot regions, such as northwest and northern Bangladesh, has diminished and gradually shifted towards the center and south. Similar findings were reported by Mohsenipour et al.^12^. Projections until the end of the century (the 2070s) anticipate a decrease in maximum drought intensity^98^. Among the three seasons in Bangladesh, the Pre-kharif season witnessed the most significant decline in drought severity over the years. Comparable changes in drought patterns have been reported periodically in several Asian countries, including Nepal, Bhutan, Cambodia, Lao PDR, India, China, and Pakistan^1,46,97,99–101^.

Considering land use changes in drought prediction models is essential for a comprehensive understanding of drought dynamics. It enables improved assessments of water availability, vegetation responses, human-induced vulnerabilities, feedback mechanisms, and the design of effective adaptation and mitigation strategies^102,103^. By accounting for land use changes, enhance the accuracy and relevance of drought predictions, ultimately supporting sustainable water resource management and resilience to drought events.

Changes in drought duration and recurrent nature justified the trends in crop damage and lower cropping intensity in Bangladesh^8^. The Climate Change Cell of Bangladesh^104^ reported that droughts reduced T. Aman rice yields by 45–60% and Rabi crop yields by 50–70%. Boro (grown in the Rabi season), the major rice, is entirely dependent on irrigation. Prolonged water scarcity encourages excessive groundwater extraction for irrigation, which further depletes groundwater levels in drought-prone regions^9^. To cope with drought, many farmers have chosen drought-resistant rice varieties cultivation^105,106^, but adoption rates remain low^107^. Other adaptation strategies included switching farming practices or changing the crop-sowing windows. Depending on the severity of the drought, some rural households may choose to migrate or change their livelihoods^108^. Overall, this could severely impact food supplies and endanger food security.

The economic losses associated with severe drought in specific countries can vary widely depending on factors such as the severity of the drought, the country's economic resilience, and its dependence on agriculture and water resources^109^. The United States experienced substantial economic losses from drought, particularly in agricultural states such as California and Texas. Prolonged droughts can lead to reduced crop yields, increased irrigation costs, and even water shortages for urban areas. The economic impact can run into billions of dollars^110^. Australia has a history of severe droughts, which can devastate the agriculture sector. The "Millennium Drought" in the early 2000s, for example, resulted in significant economic losses, affecting everything from livestock farming to wine production^111^. Severe droughts in South Africa have led to water scarcity and reduced agricultural productivity. In recent years, the country has experienced drought-related losses in key sectors like maize production and livestock farming^112^. India, with its large agricultural sector, is highly vulnerable to drought. Severe droughts can lead to crop failures, food shortages, and economic hardships for farmers. The economic losses can be substantial^113^. Droughts in Brazil impacted its important agricultural and livestock sectors. The country's economy is closely tied to these industries, making drought-related losses a significant concern^114^. In countries with fragile economies like Somalia, severe drought had devastating effects, leading to food shortages, loss of livestock, and economic distress^115^. Ethiopia experienced recurring droughts, leading to food insecurity and economic challenges. Efforts to mitigate the impact of drought and build resilience are ongoing in the country^116^. In sum, the imperative to prioritize drought mitigation cannot be overstated for nations grappling with the profound impacts of water scarcity. Making drought a top policy concern is paramount to developing comprehensive strategies encompassing preparedness, response, and recovery. As evident in the experiences of these countries, a forward-looking policy framework is crucial to building resilience, ensuring effective response mechanisms, and facilitating a swift recovery from the complexities posed by drought. By placing drought mitigation at the forefront of policy agendas, nations can proactively address the multifaceted challenges posed by water scarcity, safeguarding their economies, food security, and overall well-being for a more sustainable and resilient future.

The potential for future research on long-term drought prediction is vast and holds great significance in understanding and mitigating the impacts of drought events. In this regards, deep learning models, specifically transformers, could be used on drought prediction. Developing and refining climate models to better capture long-term climate variability and teleconnections can enhance our ability to predict droughts beyond seasonal time scales^117^. Incorporating more accurate representations of physical processes, feedback mechanisms, and regional climate dynamics can improve model performance for longer-term predictions^118^. Incorporating data from remote sensing, ground-based observations, and hydrological models can enhance our understanding of the complex feedbacks between the land surface and the atmosphere, enabling better predictions of long-term drought conditions^119^. Ensemble methods, data assimilation techniques, and hybrid models that combine the strengths of physical models and data-driven approaches may improve the skill and reliability of long-term drought predictions^120,121^. So, the understanding of long-term drought prediction and contribute to more effective planning, preparedness, and management of drought events at longer time scales is required.

## Conclusions and policy recommendations

The objective of this research is to determine the most effective machine learning methods and categorize the key factors influencing drought prediction. Utilizing a dataset spanning four decades of different weather variables, we conducted extensive statistical analysis and employed data visualization techniques to gain insights from the data. We explored a total of twenty-four machine learning models, encompassing both regression and classification approaches, which included linear, non-linear, and ensemble learning techniques. An inclusive assessment of the twenty-four models’ performance, by applying statistical metrics such as the R^2^, RMSE, and MAE method during the model validation phase. Results shows, four best ML methods, ranger, bagEarth, support vector machine, and random forest have been identified for the prediction of multi-scale drought indices. Our findings revealed that Bangladesh experienced varying levels of drought, ranging from moderate to severe and existing the shifting tendency by regions with specific deficits in average rainfall over different time periods. For instance, the deficit was 92, 95, 115, and 143 mm over 1, 3, 6, and 12-months’ time span, respectively. Temperature was identified as a crucial factor influencing drought conditions, with minimum, maximum, and mean temperatures exceeding certain thresholds resulting in severe drought conditions across different time spans. This information is valuable for understanding how temperature variations can impact drought severity. Additionally, we observed that extended periods of sunshine hours and high relative humidity levels also contributed to drought conditions in the region. Low wind speeds and high evapotranspiration further exacerbated drought conditions.

Ranking of multi-model machine learning algorithms indicate the appropriate selective options for different stations getting best prediction accuracy during drought forecasting. Reliable and effective strategies for choosing the right weather variables and constructing accurate predictive models can help to reduce the destructive impacts of drought. In this study, the application of ML models confirmed the efficacy and reliability of the research. Moreover, model selection for each region improves the study performance, and the output can be used to assess drought risk in Bangladesh. Our research proposes specific recommendations to address various aspects of drought mitigation in Bangladesh. To enhance water resource management, we advocate for strategies that improve water storage, allocation, and distribution systems, including the construction of reservoirs, promotion of water recycling and rainwater harvesting, and implementation of water conservation practices. In terms of promoting agricultural resilience, we highlight the importance of adopting climate-smart agricultural practices such as cultivating drought-tolerant crop varieties, implementing efficient irrigation techniques, practicing crop rotation, promoting agroforestry, and adopting sustainable land management practices. Additionally, we emphasize the significance of strengthening early warning systems by integrating climate data, remote sensing technologies, and advanced modeling techniques to improve the accuracy and lead time of drought predictions. Furthermore, our recommendations aim to enhance drought preparedness and response through the development of drought contingency plans, establishment of drought monitoring and assessment frameworks, and provision of financial and technical support to vulnerable communities. Lastly, we advocate for the formulation and implementation of policies that prioritize drought risk reduction and sustainable water resource management, including the integration of drought mitigation strategies into national and regional development plans, establishment of regulatory frameworks, and allocation of financial resources for drought resilience projects. By including these specific recommendations, our study aims to bridge the gap between research findings and practical applications, providing policymakers and stakeholders with tangible guidance to effectively mitigate the impacts of droughts in Bangladesh.

Future research should seek to better understand the effects of climate change on drought intensity, impact mechanism on crop, and explore strategies for adapting to longer-term drought. Collaboration between researchers, local authorities, and community organizations can help to identify effective solutions that take into account local socio-economic contexts and water management strategies that are amenable to research. Additionally, more research is needed to assess mitigation strategies, such as better water infrastructure and conservation efforts, to lessen the impacts of droughts.

### Supplementary Information

Supplementary Information.

## Data Availability

The datasets used and/or analysed during the current study available from the corresponding author on reasonable request.

## Code availability

The study primarily used the following R packages: "caret" and "ggplot2" of programming R version 4.2.1.
