# Supplementary material for: Identification of influential weather parameters and seasonal drought prediction in Bangladesh using machine learning algorithm
Source: Sci Rep. 2024 Jan 4;14:566. doi: 10.1038/s41598-023-51111-2 (PMC10767098; doi:10.1038/s41598-023-51111-2)
Supplement: Supplementary file 1 — Supplementary Information. [file 41598_2023_51111_MOESM1_ESM.docx]

**Appendix**

**Identification of influential weather parameters and seasonal drought prediction in Bangladesh using machine learning algorithm**

Md. Abdullah Al Mamun^a^, Mou Rani Sarker^b^, Md Abdur Rouf Sarkar^c,d,^*, Sujit Kumar Roy^e^, Sheikh Arafat Islam Nihad^f^, Andrew M. McKenzie^g^, Md. Ismail Hossain^a^, Md. Shahjahan Kabir^h^

***Corresponding author email address:** [mdrouf_bau@yahoo.com](mailto:mdrouf_bau@yahoo.com) (Md Abdur Rouf Sarkar)


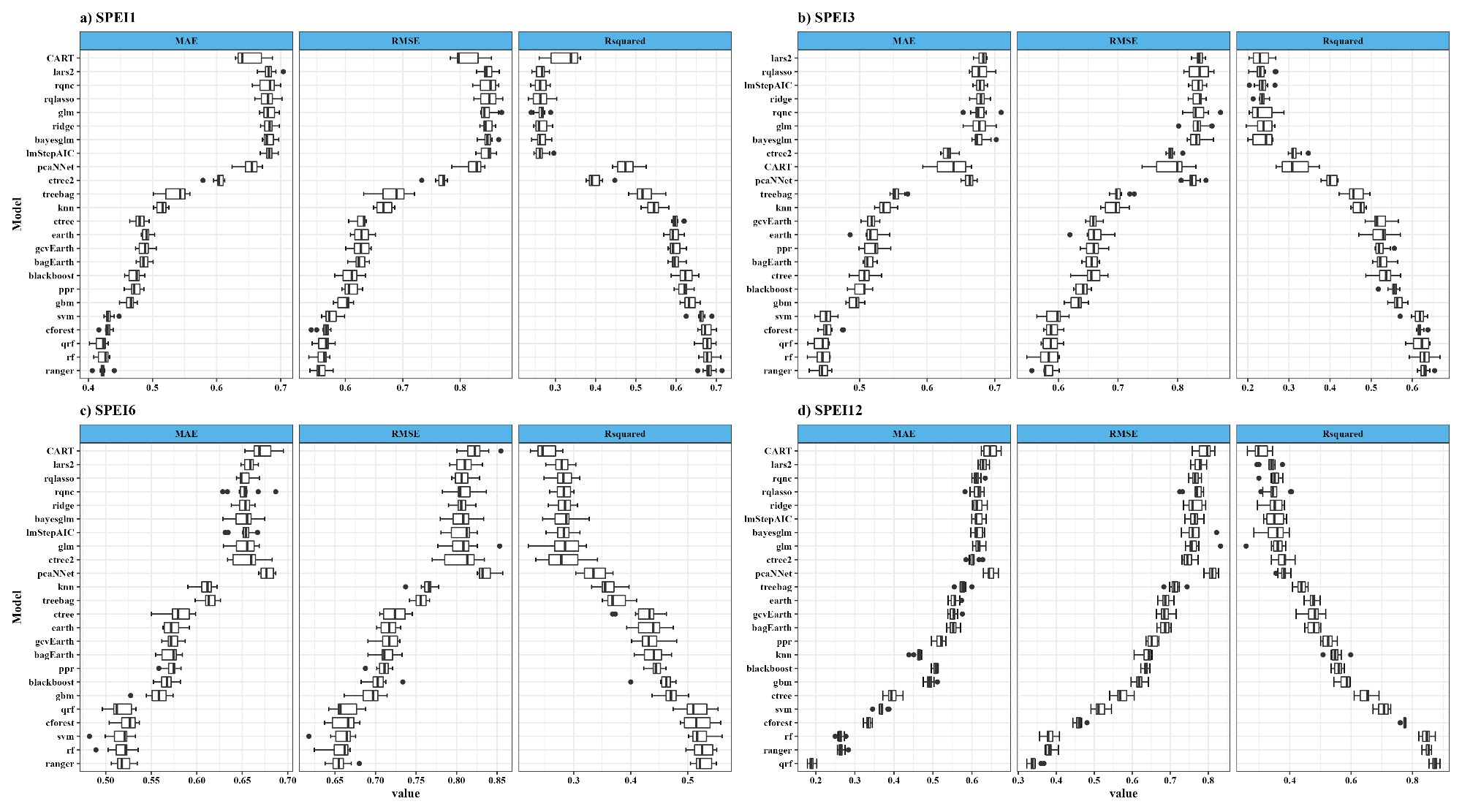


Fig. A1. Assessment criteria of ML models for predicting SPEIs during training phase for countrywide datasets. (a) SPEI1, (b) SPEI3, (c) SPEI6, and (d) SPEI12.


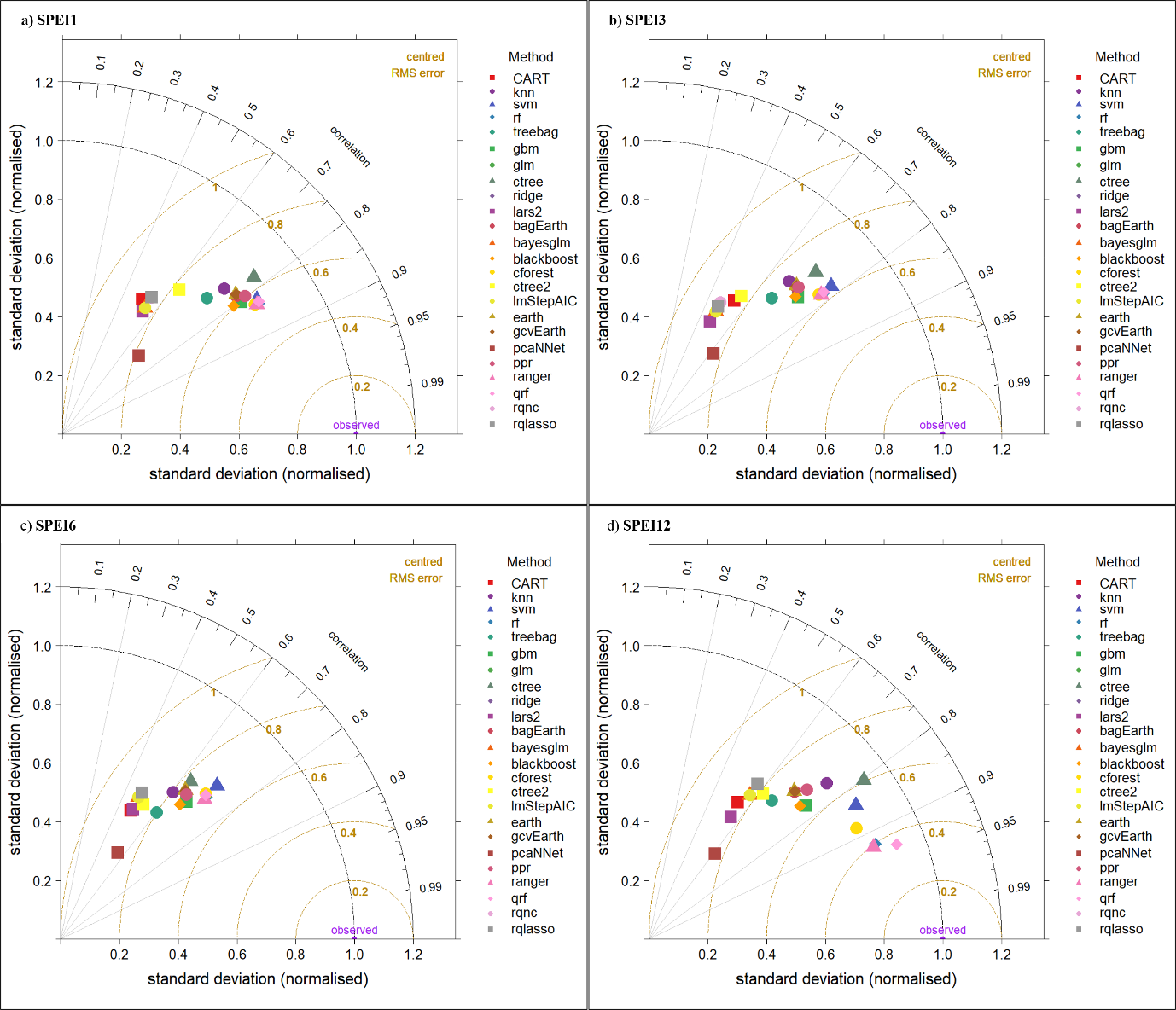


Fig. A2. Taylor diagram represents the performance of the predicted models at multiple SPEIs time scales. (a) SPEI1, (b) SPEI3, (c) SPEI6, and (d) SPEI12.


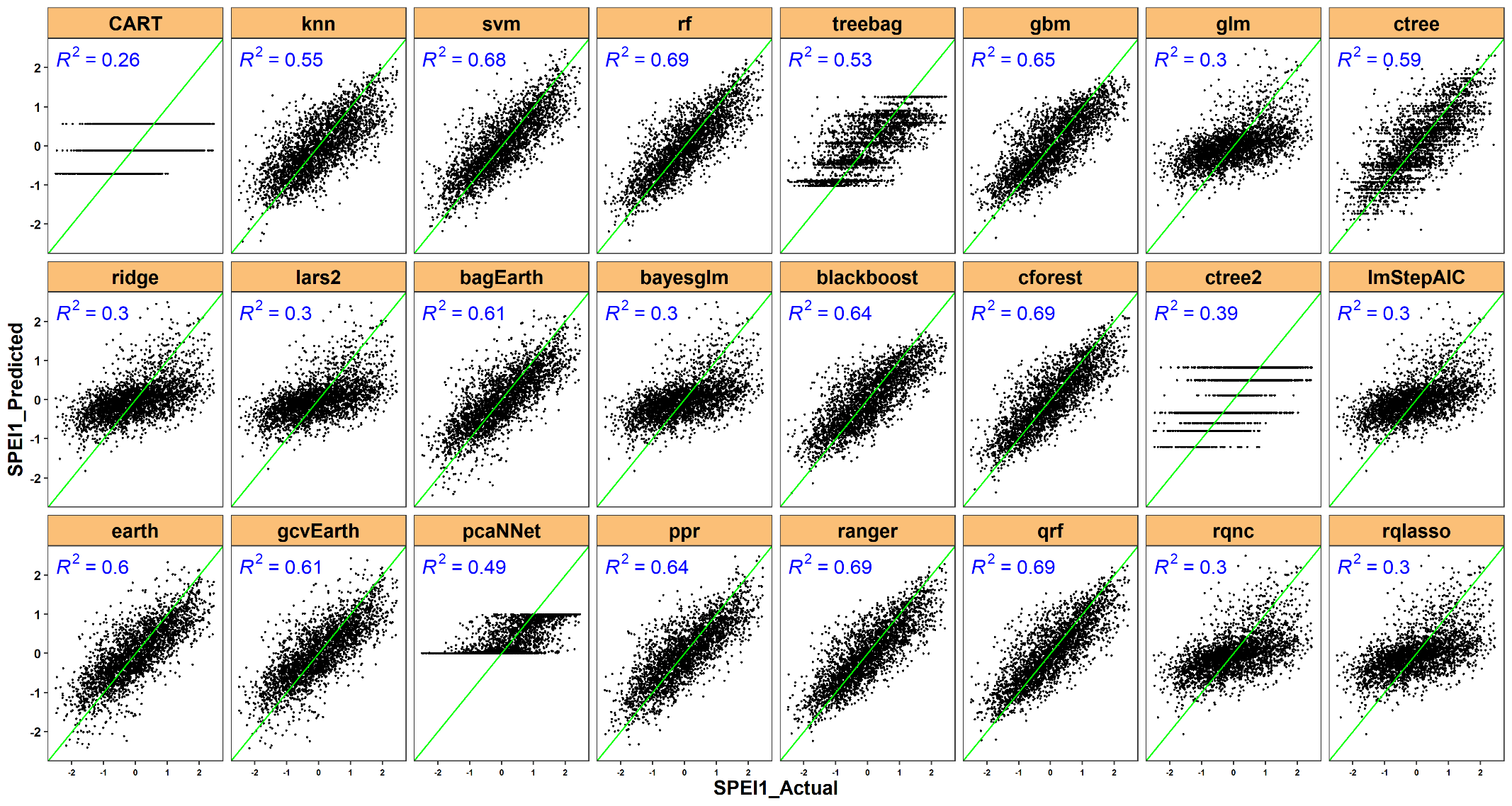


Fig. A3a. Scatter plot between the observed and predicted SPEI obtained using the twenty-four predictive ML models at SPEI1 time scales.


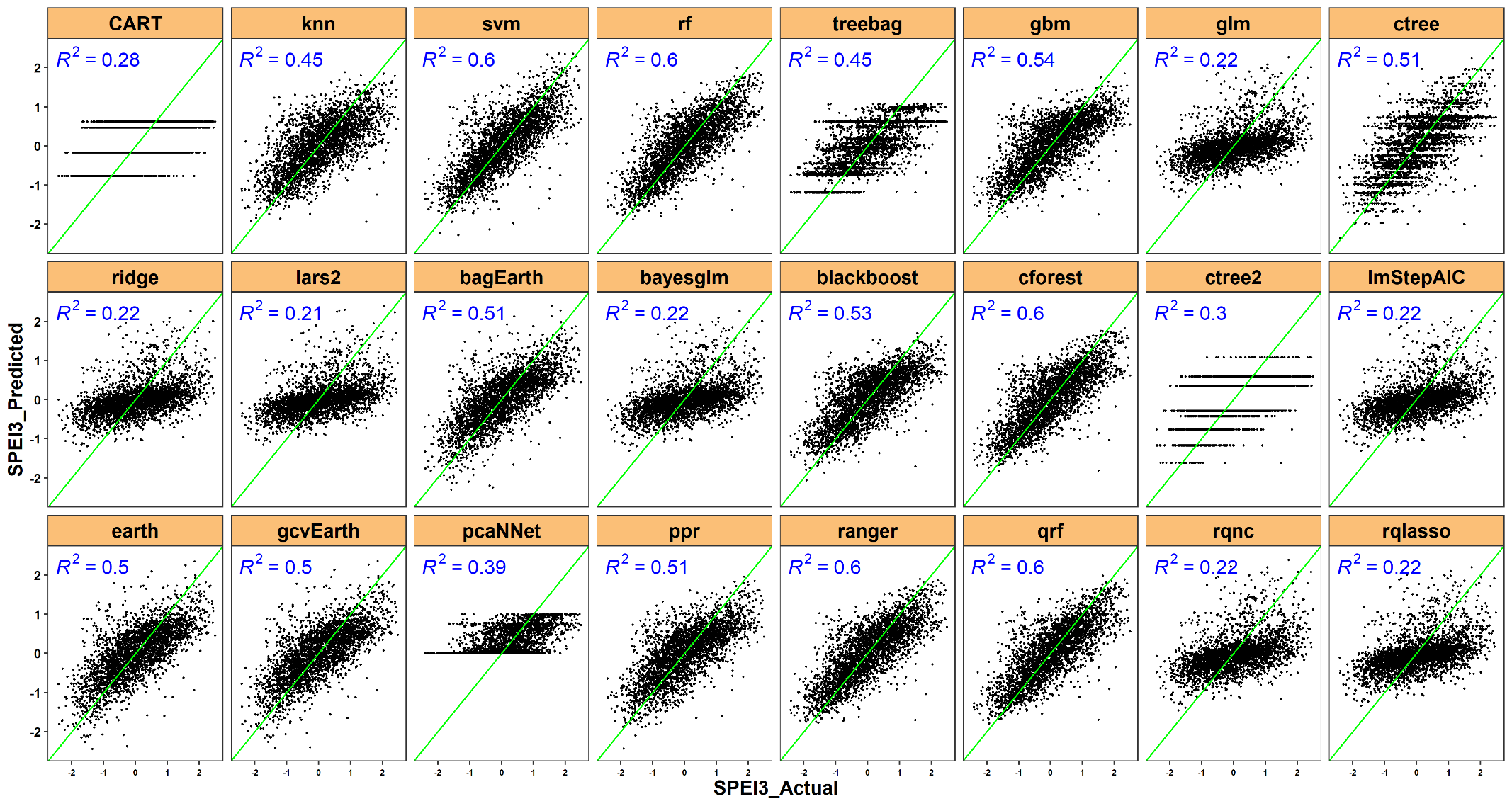


Fig. A3b. Scatter plot between the observed and predicted SPEI obtained using the twenty-four predictive ML models at SPEI3 time scales.


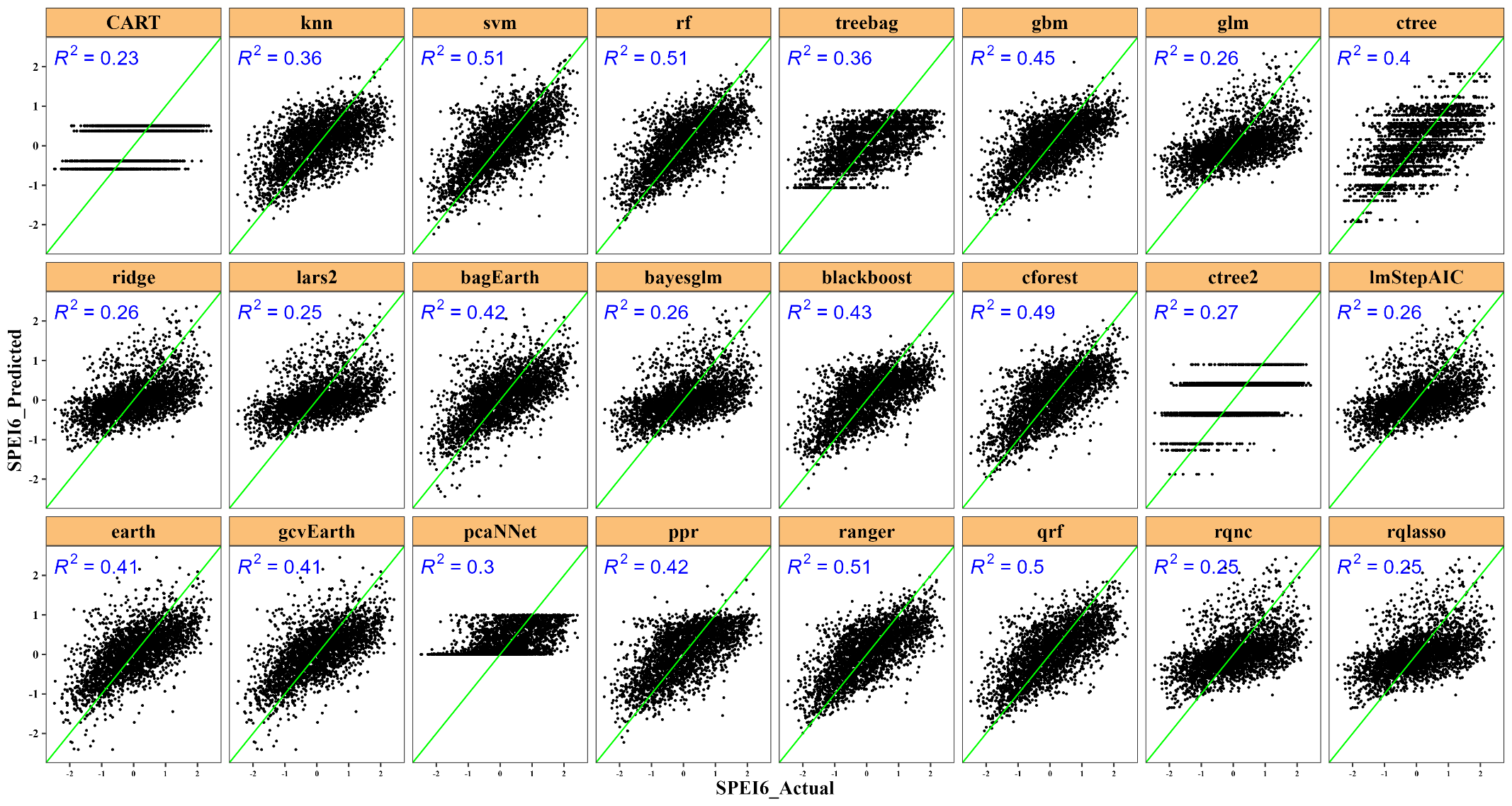


Fig. A3c. Scatter plot between the observed and predicted SPEI obtained using the twenty-four predictive ML models at SPEI6 time scales.


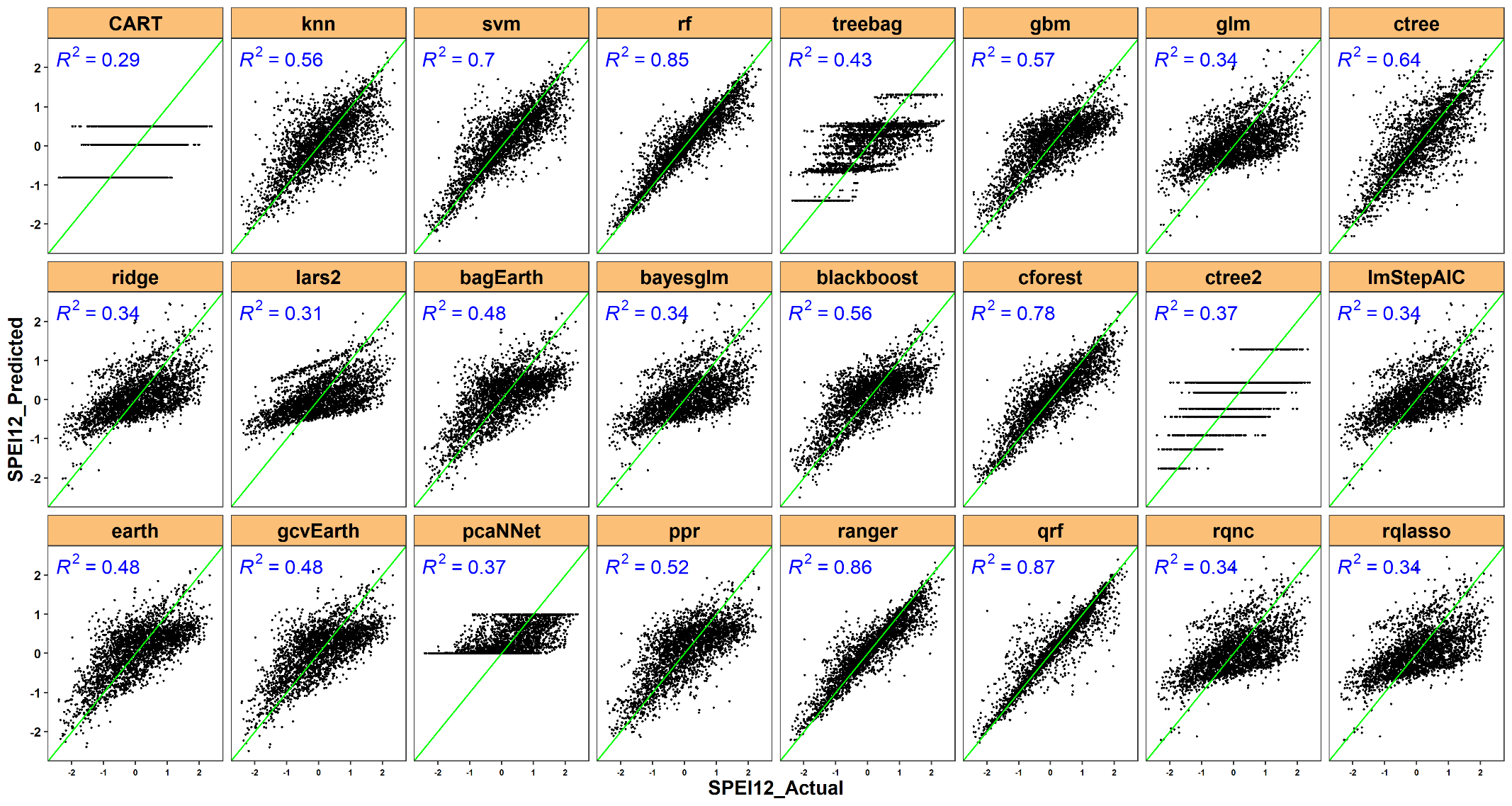


Fig. A3d. Scatter plot between the observed and predicted SPEI obtained using the twenty-four predictive ML models at SPEI12 time scales.


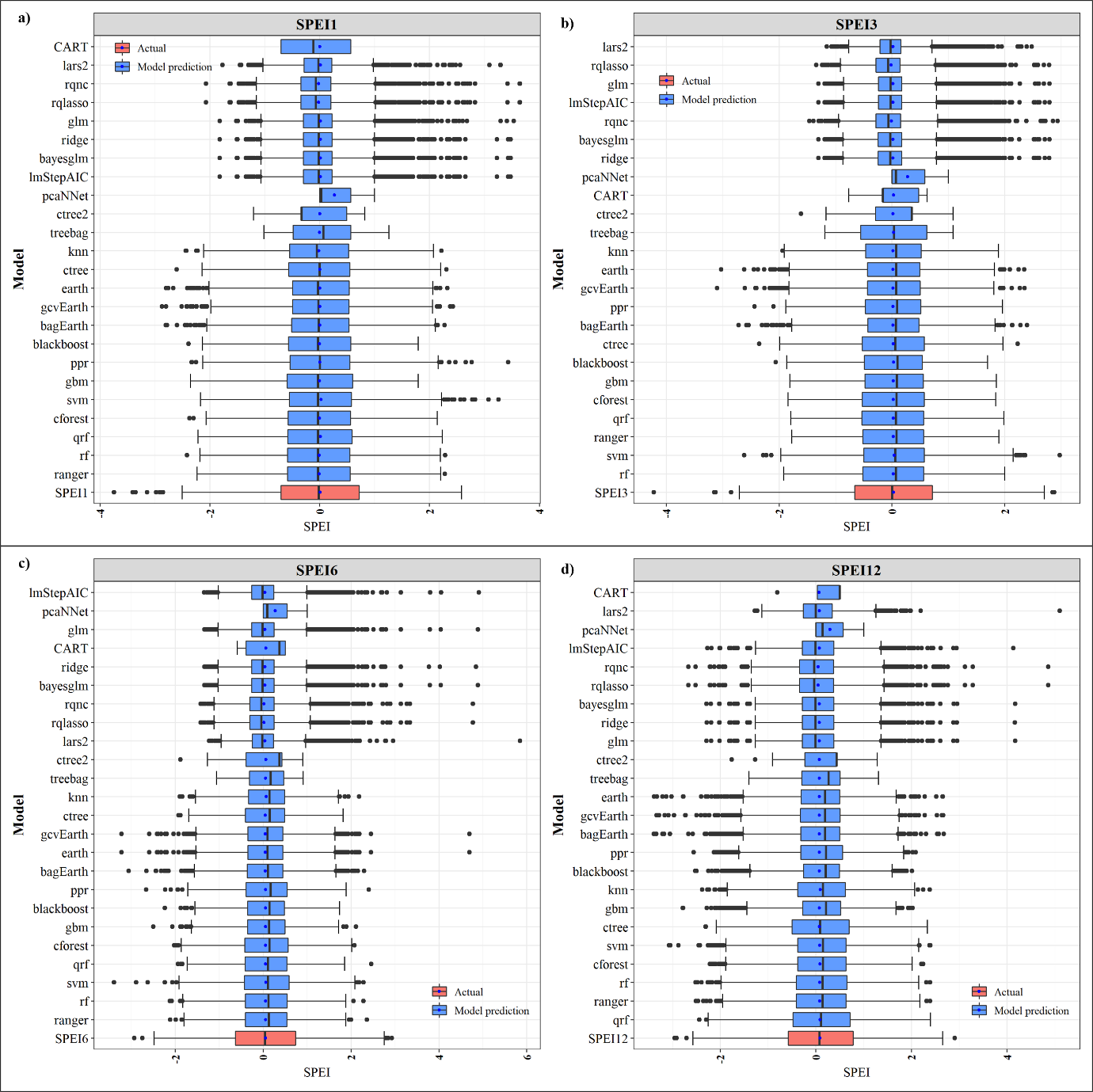


Fig. A4. Box plot visualization of prediction model performance for the four examined SPEI time scales. (a) SPEI1, (b) SPEI3, (c) SPEI6, and (d) SPEI12.
